# Naive and memory CD4^+^ T cell subsets can contribute to the generation of human Tfh cells

**DOI:** 10.1016/j.isci.2021.103566

**Published:** 2021-12-03

**Authors:** Raphaël Jeger-Madiot, Romain Vaineau, Maud Heredia, Nicolas Tchitchek, Lisa Bertrand, Mathias Pereira, Océane Konza, Bruno Gouritin, Bénédicte Hoareau-Coudert, Aurélien Corneau, Catherine Blanc, Eric Savier, Pierre Buffet, Adrien Six, David Klatzmann, Arnaud Moris, Stéphanie Graff-Dubois

**Affiliations:** 1 Sorbonne Université, INSERM, UMRS 959, Immunology-Immunopathology-Immunotherapy (i3), Paris, France; 2 Sorbonne Université, INSERM, CNRS, Center for Immunology and Microbial Infections, Paris, France; 3 Université Paris-Saclay, CEA, CNRS, Institute for Integrative Biology of the Cell, Gif-sur-Yvette, France; 4 Sorbonne Université, INSERM UMS037 PASS, Cytometry facility (CyPS), Paris, France; 5 Assistance Publique-Hôpitaux de Paris (AP-HP), Pitie-Salpetriere Hospital, Department of Hepato-Biliary and Pancreatic Surgery and Liver Transplantation, Paris, France; 6 Sorbonne Université, INSERM, St Antoine Research Center CRSA, Paris, France; 7 Université de Paris, INSERM, UMRS 1134, Biologie Intégrée du Globule Rouge, Paris, France; 8 Assistance Publique-Hôpitaux de Paris (AP-HP), Pitié-Salpêtrière Hospital, Biotherapy and Département Hospitalo-Universitaire Inflammation-Immunopathology-Biotherapy (i2B), Paris, France

**Keywords:** Immunology, Virology, Cell biology

## Abstract

CD4^+^ T follicular helper cells (Tfh) promote B cell maturation and antibody production in secondary lymphoid organs. By using an innovative culture system based on splenocyte stimulation, we studied the dynamics of naive and memory CD4^+^ T cells during the generation of a Tfh cell response. We found that both naive and memory CD4^+^ T cells can acquire phenotypic and functional features of Tfh cells. Moreover, we show here that the transition of memory as well as naive CD4^+^ T cells into the Tfh cell profile is supported by the expression of pro-Tfh genes, including transcription factors known to orchestrate Tfh cell development. Using this culture system, we provide pieces of evidence that HIV infection differentially alters these newly identified pathways of Tfh cell generation. Such diversity in pathways of Tfh cell generation offers a new framework for the understanding of Tfh cell responses in physiological and pathological contexts.

## Highlights

• *In vitro* stimulation of human spleen cells leads to the generation of Tfh-like cells
• Splenic naive and memory CD4+ T cells can acquire Tfh cell functions
• Specific programs of differentiation lead to the acquisition of Tfh cell functions
• *In vitro* HIV infection differentially alters Tfh transcriptomic programs

## Graphical abstract

**Figure:**
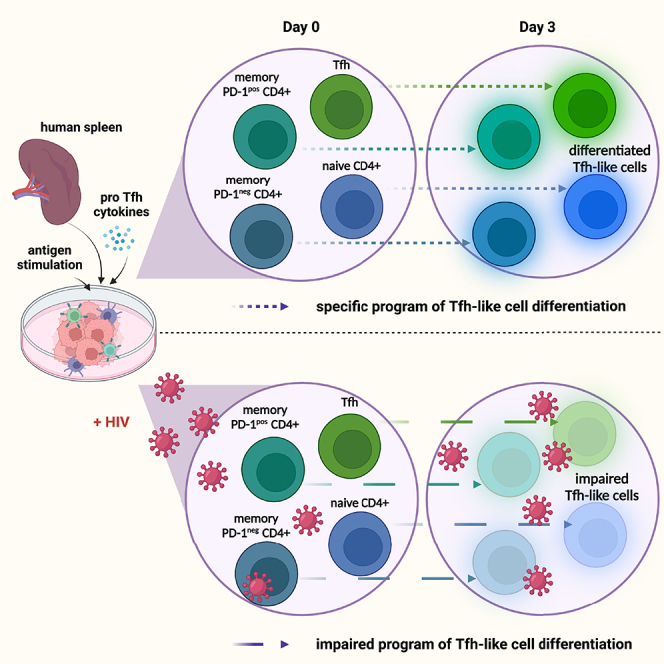

## Introduction

Within germinal centers (GCs), T follicular helper cells (Tfh) shape B cell responses by promoting the development of high-affinity antibodies, isotypic switch, and B cell maturation (Crotty, 2019; Song and Craft, 2019). Tfh cells are classically identified in secondary lymphoid organs by the expression of CXCR5 and PD-1, which drive their positioning in these lymphoid organs (Sayin et al., 2018). The establishment of the Tfh phenotype is orchestrated by the transcription factor Bcl6 (Choi et al., 2020). To control B cell maturation and GC maintenance, Tfh cells express costimulatory molecules including CD40L and ICOS and secrete cytokines such as IL-21 and IL-4 (Crotty, 2019). Until recently, Tfh cell generation was mostly considered as a sequential process where Tfh cells arise after naive CD4^+^ T cell priming by dendritic cells in the T cell zone and acquisition, in the B cell zone, of fully effective functions following cognate interactions with B cells. However, several *in vitro* experiments have shown that memory CD4^+^ T cells can acquire Tfh cell features upon stimulation (Jacquemin et al., 2015; Lu et al., 2011; Pattarini et al., 2017). Thus, heterogeneous Tfh cell profiles might result from cellular plasticity in CD4^+^ T cell populations in lymphoid tissues.

Deciphering the various pathways leading to Tfh cell generation is of particular interest in chronic infectious diseases such as HIV where a paradoxical increase of dysfunctional Tfh cells has been reported (Colineau et al., 2015; Lindqvist et al., 2012; Perreau et al., 2013). As HIV infection is associated with architectural alterations of lymphoid tissues and CD4^+^ T cell exhaustion, we hypothesized that increase of Tfh could result from the unregulated reprogramming of CD4^+^ T cells into Tfh in lymphoid organs that sustain viral antigenic stimulation (Jeger-Madiot et al., 2019).

Addressing pathways of Tfh cell generation remains challenging in humans. Until recently, systems relying on lymphoid cell suspensions were mainly used to study the spread of HIV infection and the development of Tfh was not addressed.

Assuming that lymphoid cell cooperation synergizes to generate Tfh, we developed an original culture system based on the stimulation of splenic mononuclear cell suspensions. Using this strategy, we obtained a robust Tfh cell-like response including both non-GC and GC Tfh, as opposed to the use of peripheral blood mononuclear cells (PBMCs) which did not lead to generation of GC Tfh. Thanks to flow and mass cytometry combined with bulk RNA sequencing, we found that naive and memory CD4^+^ T cell subsets could differentiate toward a Tfh cell profile. Most importantly, the gain of Tfh cell phenotype by both naive and memory CD4^+^ T cell subsets was associated with specific transcriptional reprogramming. The reprogramming of various CD4^+^ T cell subsets leads to distinct phenotypes of Tfh, with differential expression of co-stimulatory molecules and cytokine secretion. As the impact of HIV infection on Tfh cell polarization was never addressed in a system involving a global lymphoid microenvironment, we investigated it using our original model. We showed that *in vitro* HIV infection modulates the acquisition of a Tfh cell profile by naive and memory CD4^+^ splenocyte subsets. Taken together, our results indicate that the heterogeneity of Tfh cell responses likely reflects the differential contribution of several CD4^+^ T cell subsets to the Tfh cell pool. Our work provides a framework for a better understanding of human Tfh cell biology under physiological and pathogenic conditions.

## Results

### Antigen-experienced splenocytes lead to the generation of Tfh

**Figure 1:**
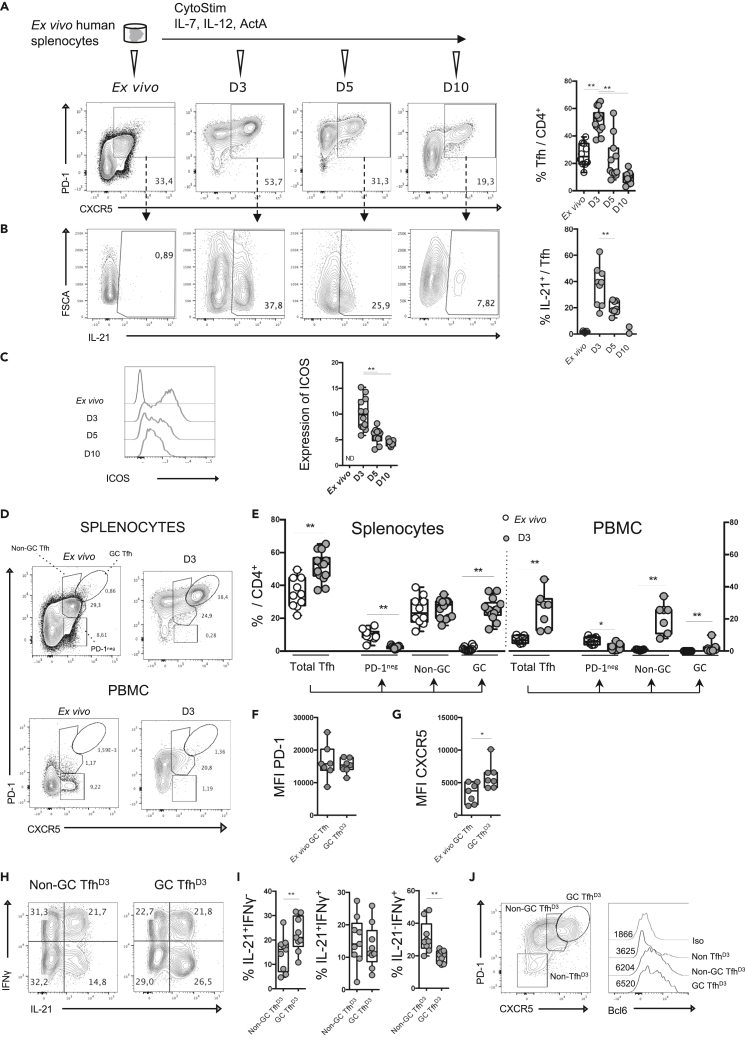
Antigenic stimulation of human splenic mononuclear cells mimics the T CD4^+^ response of GC reaction (A) Splenic mononuclear cells (splenocytes) were stimulated with CytoStim and cultured for 3 days in the presence of cytokines (IL-7, IL-12, activin) (A). CXCR5 and PD-1 expressions among CD4+ T cells were assessed. Representative flow plots showing CXCR5 and PD-1 expression on CD4^+^ T cells from *ex vivo* splenocytes and splenocytes cultured for 3, 5, and 10 days (left) and the percentage of Tfh among CD4+ T cells (right). (B) Representative flow plots showing IL-21 production by Tfh (left) and the percentage of IL-21-positive cells among Tfh (right). (C) Representative histogram showing ICOS expression among Tfh (left) and relative expression of ICOS among Tfh (right). (D) Gating strategy allowing the identification of PD-1^neg^ Tfh, non-GC Tfh, and GC Tfh among total CXCR5^+^PD-1^+^ cells *ex vivo* or after 3 days of stimulation of splenocytes or PBMCs in polarizing cytokines. (E) Percentage of total CXCR5^+^PD-1^+^ cells including PD-1^neg^ Tfh, non-GC Tfh, and GC Tfh among CD4^+^ T cells *ex vivo* or after 3 days of culture using splenocytes or PBMCs (n = 7–14). (F and G) Mean fluorescence intensity of PD-1 (F) and CXCR5 (G) expression on *ex vivo* GC Tfh and GC Tfh^D3^ splenic cells. (H) Representative flow plots showing IL-21 and IFNγ production by non-GC Tfh^D3^ and GC Tfh^D3^ cells 3 days after splenocyte stimulation. (I) Percentage of IL-21- and/or IFNγ-positive cells among non-GC Tfh^D3^ cells and GC Tfh^D3^. (J) Gating strategy for analysis of Bcl6 expression in CD4^+^ T cells and histograms showing Bcl6 mean fluorescence intensity for GC Tfh^D3^, non-GC Tfh^D3^, and CXCR5^−^PD-1^-^ CD4^+^ T cell subsets (n = 14). Each symbol represents an individual donor. A Wilcoxon matched pairs test was performed; ∗, p < 0.05; ∗∗, p < 0.005.

As Tfh differentiate in the specific environment of lymphoid organs, we hypothesized that generation of Tfh would be optimized using splenic mononuclear cell suspensions. Cell suspensions from healthy donors were stimulated using CytoStim (Miltenyi), which acts as a T cell superantigen by cross-linking the T cell receptor and MHC molecules. Cells were then cultured for 10 days with IL-7, IL-12, and activin A (Figure 1A), which are reported to be enhancers of Tfh cell generation (Carnathan et al., 2020; Durand et al., 2019; Locci et al., 2016). By evaluating the expression of CXCR5 and PD-1 on CD4^+^ T cells over time, we found that, after 3 days, the proportion of CXCR5^+^ PD-1^+^ Tfh among CD4^+^ T cells was doubled and then started to decline to reach 10% at day 10 (Figure 1A). Moreover, the proportion of IL-21-producing cells and cells expressing ICOS among induced Tfh follows the same kinetics, suggesting that cells induced after splenocyte stimulation acquire Tfh cell functional features (Figures 1B and 1C). Of note, the proportion of live-dead stained cells among CD4^+^ T cells did not increase between day 3 (D3) and day 5 (D5), suggesting that the decrease of Tfh results from a return to a resting state rather than from cell death (Figure S1A). Finally, Tfh were not more prone to cell death compared with other activated CD4+ T cells (CXCR5^−^PD-1^+^) (Figure S1B) on day 3.

Then, we evaluated our stimulation protocol on PBMCs to test its capacity to promote Tfh in a non-lymphoid environment. To better characterize induced Tfh we distinguished GC Tfh, which expresses high levels of CXCR5 and PD-1, from non-GC Tfh (Haynes et al., 2007; Sayin et al., 2018; Vella et al., 2019). First, *ex vivo* circulating Tfh cell staining showed that total Tfh represented 7.1% (±2.2) of CD4^+^ T cells and were mainly PD-1^neg^, while splenocytes showed 26.6% of PD-1^pos^Tfh including 1.5% (±1.3) of GC Tfh. By submitting PBMCs to our stimulation protocol, we observed that Tfh expanded from 7.1% to 23.3% between D0 and D3 without giving rise to GC Tfh. These results suggested that GC Tfh cell generation was restricted to splenocyte stimulation (Figures 1D and 1E) where GC Tfh^D3^ express similar levels of PD-1 compared with *ex vivo* GC Tfh and higher expression of CXCR5 (Figures 1F and 1G). Interestingly, the generation of GC Tfh was reproduced using lymph node mononuclear cell suspensions (not shown). Such an increase of GC Tfh using lymphoid mononuclear cell stimulation strongly suggests that an activated lymphoid environment supports a complete Tfh cell response. Thus, the opportunity to examine Tfh cell biology appears more relevant with the stimulation of mononuclear cells from lymphoid organs. As reported for GC Tfh and non-GC Tfh from tonsils (Brenna et al., 2020), we found that GC Tfh^D3^ were preferentially associated with IL-21 secretion, while their frequency was reduced among IFN-secreting cells (Figures 1H and 1I). Consistently, Bcl6 expression was much greater in both GC Tfh^D3^ cells and non-GC Tfh^D3^ compared with non-Tfh (Figure 1J).

To better characterize the signals required for Tfh cell generation in our system, we modified the protocol by variating CytoStim stimulation and cytokines. First, CytoStim was required to induce *de novo* Tfh (not shown) and to generate GC Tfh (Figures S1C and S1D), showing that a sustained T cell receptor signal is needed for the generation of GC Tfh, as previously reported (Baumjohann et al., 2013). By modulating the cytokine environment, we found that addition of exogenous cytokines greatly enhanced GC Tfh^D3^ proportions (Figures S1C and S1D). Moreover, the addition of the cytokine cocktail was required to induce expression of IL-21 and ICOS (Figures S1E and S1F). Hence, using splenocytes, we designed a reproducible experimental design that supports the establishment of fully differentiated Tfh, with GC Tfh cell generation peaking after 3 days of culture.

Finally, we questioned the capacity of GC Tfh^D3^ to promote the maturation of CD27^hi^CD38^hi^ plasma cells. To this end, GC Tfh^D3^ and CXCR5^−^PD-1^+^ CD4^+^ T^D3^ cells were sorted and co-cultured with autologous CD19^+^ B cells to induce their maturation. After 7 days, the proportion of CD27^hi^CD38^hi^ plasma cells revealed that GCTfh^D3^ sustained plasma cell differentiation more efficiently than activated CD4^+^CXCR5^−^PD-1^+^ T^D3^ cells (Figures S1G and S1H). Altogether, these data indicate that GC Tfh^D3^ recapitulated phenotypically and functionally the features of activated *bona fide* Tfh. Thus, our experimental design appears suitable for studying the dynamics of Tfh cell responses to antigenic stimulation in lymphoid tissue.

### *Ex vivo* and induced Tfh display distinct phenotypic landscapes and differentiation trajectories

**Figure 2:**
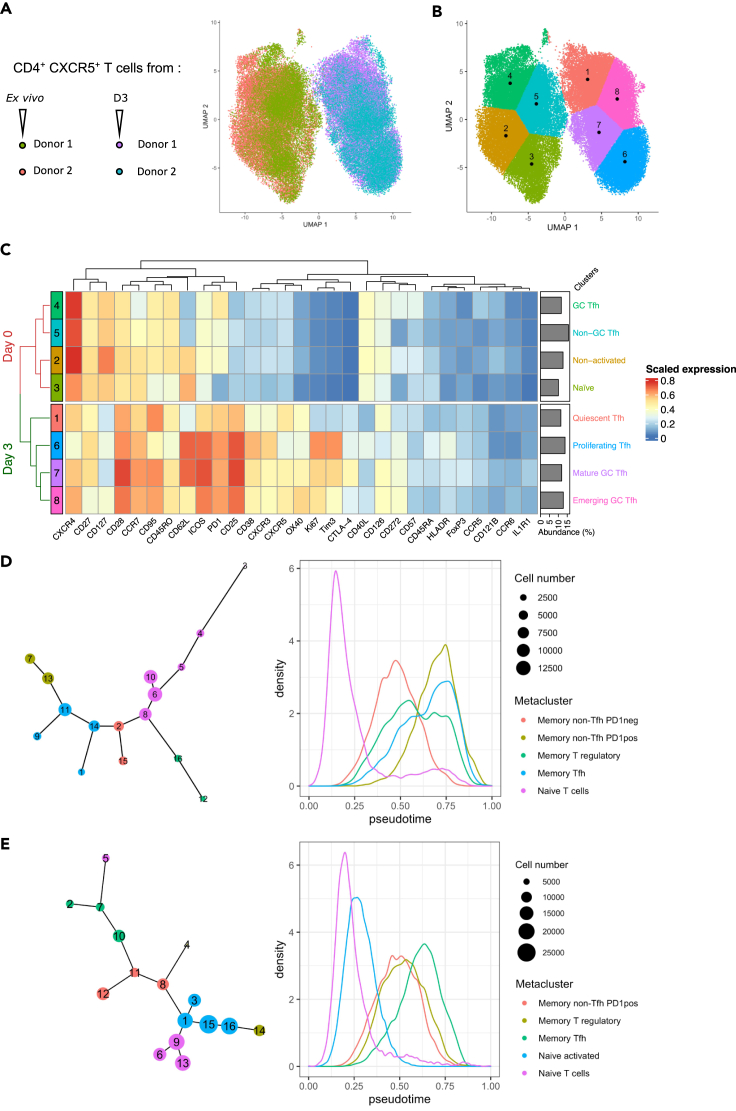
*Ex vivo* and induced Tfh display distinct phenotypic landscapes and differentiation trajectories (A) Deep immunophenotyping was performed after 3 days of culture (n = 2 independent donors). CD4^+^ CXCR5^+^ T cell selection was based on the expression of CD45, CD8, CD11c, CD56, and CD19. The Uniform Manifold Approximation and Projection (UMAP) algorithm was used to represent the whole set of CD4^+^ CXCR5^+^ T cells in a multiparametric manner at D0 and D3 after antigenic stimulation of splenocytes from two donors. (B) Projection of 8 clusters determined by k-means on the UMAP representation of D0 and D3 CD4^+^ CXCR5^+^ T cells. (C) Heatmap representing the mean expression of 29 markers by 8 cell clusters and their relative abundance, defined among D0 and D3 CD4^+^ CXCR5^+^ T cells. (D and E) Trajectory and pseudotime analysis on total D0 (D) and D3 (E) CD4^+^ T cells. Tree plots (left) show cluster trajectories, cell number, and metacluster assignment, and density plots (right) show the density of pseudotime across metaclusters.

To further investigate the landscape of *ex vivo* splenic Tfh and the phenotypic modifications induced after splenocyte stimulation, we performed deep immunophenotyping using mass cytometry with 29 different markers related to CD4^+^ T cell biology (Table 1). We focused on *ex vivo* (D0) and D3 time points of splenocyte stimulation, the latter corresponding to the peak of Tfh cell generation. A Uniform Manifold Approximation and Projection (UMAP) representation of CD4^+^ CXCR5^+^ cells from two donors was performed. Strikingly, CD4^+^ CXCR5^+^ cells from D0 and from stimulated splenocytes (D3) clearly clustered separately, with uniform distribution of cells at each time point for the two donors (Figure 2A). In accordance with results presented in Figure 1, Tfh^D3^ displayed an activated phenotype, characterized by higher expression of activation markers (Tim3, PD-1, CD38, CD25, CD95, Ki67) and costimulatory molecules (CD28, ICOS, OX40), as compared with *ex vivo* Tfh (Tfh^D0^) (Figure S2A). These observations were confirmed by the analysis of Tfh^D3^ from two additional donors (not shown). Of note, FoxP3^+^ expression does not vary significantly between D0 and D3, indicating that the splenocyte stimulation favors the generation of helper rather than regulatory follicular T cells at day 3 after stimulation. Using the k-means algorithm, four clusters of CD4^+^ CXCR5^+^ cells could be defined at D0 (Figure 2B), namely, naive (cluster 3; CD45RA^hi^ CD45RO^lo^ PD-1^lo^ CD62L^hi^) and non-activated (cluster 2; CD45RA^int^ PD-1^int^ CD127^hi^ CD62L^hi^) CD4^+^ CXCR5^+^ cells, together with GC Tfh (cluster 4; PD-1^pos^ ICOS^pos^ CCR7^lo^ CXCR4^hi^ CD272^pos^) and non-GC Tfh (cluster 5; PD-1^int^ ICOS^pos^ CCR7^int^ CD127^pos^) cells, the latter being predominant (15.7% of total cells) (Figure 2C). Coherently, CXCR5 expression was increased from D0 to D3, together with immune checkpoint molecules (CTLA-4, Tim3), confirming that Tfh^D3^ are activated after splenocyte stimulation (Figure S2A). Although the expression intensity of chemokine and cytokine markers as CCR6, CCR5, and CD126 did not vary at D3, interestingly, expression of CXCR3, which has been associated with tonsillar Tfh, was increased (Brenna et al., 2020). Also, four clusters were identified at D3 (Figure 2B). Two similar clusters exhibited a highly functional phenotype, namely, mature GC Tfh (cluster 7; PD-1^hi^ ICOS^hi^ CD127^lo^ CXCR4^pos^ CD95^hi^ CD28^hi^ CXCR3^lo^) and emerging GC Tfh (cluster 8; PD-1^hi^ ICOS^hi^ CD127^pos^ CXCR4^hi^ CD95^hi^ CD28^hi^ CXCR3^pos^). The two other clusters corresponded to proliferating Tfh (cluster 6; Ki67^hi^ PD-1^hi^ CXCR5^int^ CD62L^hi^ CD45RO^lo^) and quiescent Tfh (cluster 6; PD-1^pos^ CD27^lo^ CD62L^lo^) (Figure 2C).

**Table 1:** CyTOF antibody panel

Label	Target	Clone	Providers
89Y	CD45	HI30	Fluidigm
141Pr	CD196 (CCR6)	11A9	Fluidigm
142Nd	CD19	HIB19	Fluidigm
143Nd	CD45RA	HI100	Fluidigm
144Nd	CD38	HIT2	Fluidigm
145Nd	CD4	RPA-T4	Fluidigm
146Nd	CD8a	RPA-T8	Fluidigm
147Sm	CD195 (CCR5)	REA245 (IgG1)	Miltenyi
148Nd	CD197 (CCR7)	REA546	Fluidigm
149Sm	CD25 (IL-2R)	2A3	Fluidigm
150Nd	CD272 (BTLA)^a^	Polyclonal (IgG)	RnD system
151Eu	CD278/ICOS	C398.4A	Fluidigm
152Sm	CD95/Fas	DX2	Fluidigm
153Er	Tim-3	F38- 2 × 10^2^	Fluidigm
154Sm	IL1R1^a^	Polyclonal (IgG)	RnD system
155Gd	CD279 (PD-1)	EH12.2H7	Fluidigm
156Gd	CD183 (CXCR3)	G025H7	Fluidigm
158Gd	CD134 (OX40)	ACT35	Fluidigm
159Tb	FoxP3	259D/C7	Fluidigm
160Gd	CD28	CD28.2	Fluidigm
161Dy	IL6Ralpha^a^	REA291	Miltenyi
162Dy	CD27	L128	Fluidigm
163Dy	CD57	HCD57	Fluidigm
164Dy	CD45RO	UCHL1	Fluidigm
165Ho	CD127 (IL-7Ra)	A019D5	Fluidigm
166Er	IL1R2^a^	34141	RnDsystems
167Er	SH2D1a^a^	782702	RnDsystems
168Er	CD154 (CD40L)	24-31	Fluidigm
170Er	CD152 (CTLA-4)	14D3	Fluidigm
171Yb	CD185 (CXCR5)	RF8B2	Fluidigm
172Yb	Ki-67	B56	Fluidigm
173Yb	HLA-DR	L243	Fluidigm
174Yb	CD56 (NCAM)^a^	REA196 (IgG1)	Miltenyi
175Lu	CD184 (CXCR4)	12G5	Fluidigm
176Yb	CD62L (L-selectin)^a^	REA615	Miltenyi
209Bi	CD11b (Mac-1)	ICRF44	Fluidigm

a Manually coupled using the Maxpar X8 Antibody Labeling Kit (Fluidigm).

In order to decipher whether induced Tfh originate from a linear differentiation of naive T cells or not, we performed a trajectory inference combined with a pseudotime analysis on total D0 and D3 CD4^+^ T cells. We identified 16 clusters at each time point (Figures S2B and S2D), and we assigned them to metaclusters following the expression of 30 markers, thus obtaining biologically relevant metaclusters (Figures S2C and S2E). After pseudotime calculation, the first striking observation was that naive T cell metaclusters were the “earliest,” whereas Tfh cell subsets were the “latest,” at both time points (Figures 2D and 2E), confirming that Tfh represent a terminal stage of CD4^+^ T cell differentiation. At D0, memory non-Tfh PD-1^pos^ and PD1^neg^ metaclusters were the closest to Tfh, suggesting that both are transitional subsets between naive T cells and Tfh. Moreover, *ex vivo* memory non-Tfh PD-1^pos^ cells did not seem to originate from naive T cells, which is coherent with resting spleens deprived of antigenic stimulation (Figure 2D). At D3, naive T cell clusters were no longer the most abundant, being replaced by activated cells (i.e., naive activated and memory subsets) (Figure 2E). At this time point, the Tfh cell metacluster seemed directly derived from both naive T cells and memory non-Tfh PD1^pos^ clusters, indicating multiple likely trajectories giving rise to early Tfh (cluster 7) and mature Tfh (cluster 10), respectively (Figures 2E and S2D). Although the subset of origin may dictate Tfh^D3^ phenotype, other subsets branched out from the global linear trajectory such as memory T regulatory cells, giving rise to either memory non-Tfh PD1^pos^-derived follicular regulatory T cells (Tfr, cluster 4) or naive activated-derived regulatory T cells (Treg, cluster 14) (Figures 2E and S2D). Finally, D3 trajectory from naive to Tfh cells remained similar to D0, with the exception of naive activated cells that emerged as an intermediate between naive and memory subsets. Overall, despite the important heterogeneity of stimulation-induced Tfh, D0 and D3 memory Tfh metaclusters shared a core group of differentially expressed markers (CXCR5, PD-1, CD57, CXCR4, CD45RO, CD95, CD126), highlighting that we were able to generate *ex vivo*-like Tfh. Taken together, these results suggest that our splenocyte stimulation protocol leads to strong induction of activated Tfh^D3^ cells either directly from naive CD4^+^ T cells or through memory PD-1^neg/pos^ CD4^+^ T intermediates.

### Naive and memory CD4^+^ T cells take different developmental pathways to become Tfh

**Figure 3:**
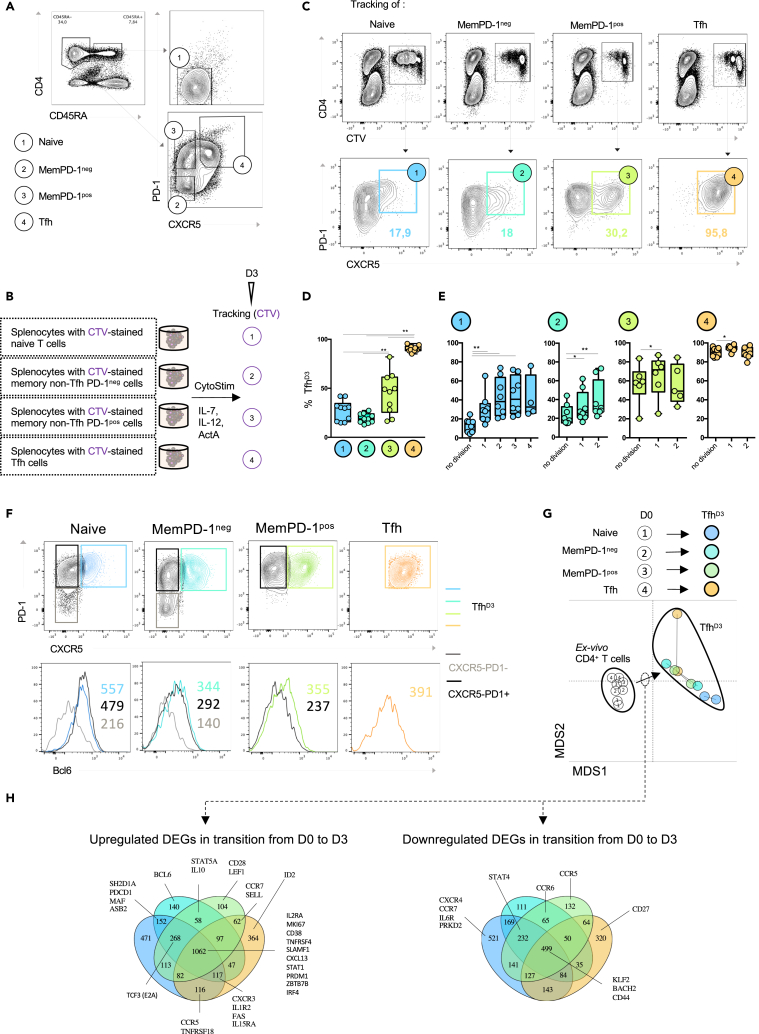
Naive and memory CD4^+^ can orient toward a Tfh cell profile (A) Four CD4^+^ T cell subsets are defined at day 0: (1) naive CD4^+^ CD45 RA^**+**^ T cells, (2) memory CD4^+^ CD45 RA^**-**^ PD-1^**-**^ T cells (MemPD-1^neg^), (3) memory CD4^+^ CD45 RA^**-**^ PD-1^**+**^ T cells (MemPD-1^pos^), and (4) Tfh. (B) At day 0, D4^+^ T cell subsets were sorted according to the gating strategy presented in (A). Isolated CD4^+^ T cell subsets were stained with cell trace violet (CTV) and mixed back into the negative splenocyte fraction. Stimulation and culture were next performed as previously described (Figure 1A). (C) Representative flow plots of CTV tracking for each stimulated CD4^+^ T cell subset 3 days after antigenic stimulation (top) combined with flow cytometry analysis of CXCR5 and PD-1 expression among CTV^+^ cells at day 3 (bottom). (D) Percentage of CXCR5^+^PD-1^+^ cells (Tfh^D3^) among traced CD4^+^ T cell subsets after 3 days of antigenic stimulation. (E) Percentage of Tfh^D3^ cells according to the divisions of stimulated CD4^+^ T cell subsets. (F) Representative histograms of Bcl6 expression for Tfh^D3^ (colored line) compared with CXCR5^−^PD-1^**+**^ (black line) and CXCR5^−^PD-1^**-**^ (gray line) deriving from respective CD4^+^ T cell subsets naive (blue), MemPD-1^neg^ (turquoise blue), MemPD-1^pos^ (green), and Tfh (orange). (G) RNA sequencing was performed on CD4^+^ T cell subsets (Day 0) and the corresponding derived Tfh^D3^ counterparts (n = 2). Multidimensional scaling was used to better visualize transcriptomic proximity of different CD4^+^ T cells. (H) Venn diagrams were used to highlight Tfh-associated genes (Table 2) among differentially expressed genes that were shared during transition from D0 CD4^+^ T cell subsets to their Tfh^D3^ counterparts (D and E). Each symbol represents an individual donor. A Wilcoxon matched pairs test was performed; ∗, p < 0.05; ∗∗, p < 0.005.

We optimized our experimental design to monitor the evolution of distinct CD4^+^ T cell subsets in the lymphoid environment. Since CD4^+^ T cell subsets exhibit phenotypic plasticity in response to environmental stimuli, we followed the dynamics of naive CD4^+^ T cells and memory non-Tfh. Two subsets of memory non-Tfh were distinguished. PD-1^pos^ memory CD4^+^ T cells (memPD-1^pos^) were defined as activated cells, whereas PD-1^neg^ (memPD-1^neg^) were defined as non-activated cells. Indeed, transcriptome analysis revealed that PD-1 expression was associated with recent T cell receptor stimulation and that memPD-1^pos^ cells displayed higher ICOS expression (Figures S3A and S3B). In addition to studying the fate of naive and memory non-Tfh, we studied the fate of *ex vivo* Tfh (Tfh^D0^), which are mainly composed of non-GC Tfh (Figure 3A). Each CD4^+^ T cell subset was isolated from whole splenocytes using flow cytometry, then labeled with Cell Trace Violet (CTV) and re-incorporated into the negative fraction of splenocytes (Figure 3B). Then, we investigated the ability of CTV-labeled CD4^+^ T cell subsets to express CXCR5 and PD-1 3 days after splenocyte stimulation (n = 10 donors). Tfh^D3^ derived from Tfh^D0^ cells were still positive for expression of CXCR5 and PD-1 markers (Figures 3C and 3D). Remarkably, 27% ± 10.4 of naive CD4^+^ T cells became Tfh after 3 days of culture. The proportion of Tfh^D3^ cells derived from memPD-1^pos^ cells was significantly higher (44.7% ± 20.9) than the proportion derived from memPD-1^neg^ cells (19.4% ± 4.8) (Figures 3C and 3D). This suggests a differential contribution of memory CD4^+^ T cell subsets to the global Tfh^D3^ pool according to their activation status.

Taking advantage of CTV staining, we further analyzed the expression of CXCR5 and PD-1 through the cell division cycles. First of all, Tfh maintained their CXCR5 and PD-1 expression through all division cycles. Second, the percentage of Tfh^D3^ cells peaked after only one division cycle for memPD-1^pos^ or Tfh^D0^ cells, whereas three and two divisions were required for memPD-1^neg^ cells and naive CD4^+^ T cells, respectively (Figure 3E). These data suggest that, compared with other CD4 T cell subsets, the higher yield of Tfh^D3^ derived from memPD-1^pos^ results more from their higher capacity to convert into Tfh than from their overproliferation.

Moreover, whatever the CD4^+^ T cell subset, Bcl6 was expressed more in Tfh^D3^ cells than in non-Tfh (CD4^+^ CXCR5^-^) derived from the same origin (Figure 3F), suggesting the induction of a transcriptional program promoting Tfh cell differentiation in each non-Tfh CD4^+^ T cell subset.

To further define whether Tfh cell phenotype acquisition was associated with a Tfh-related transcriptional program, we performed a transcriptomic analysis of *ex vivo* isolated naive, memPD-1^pos^, memPD-1^neg^ Tfh and of their respective Tfh^D3^ counterparts (n = 2 donors) (Figure 3G).

Coherently with mass cytometry analysis, Tfh^D0^ and Tfh-derived Tfh^D3^ clustered apart. Indeed, by comparing the secreting capacities of Tfh^D0^ with those of Tfh^D3^, we evidenced a great increase in IL-21 secretion, confirming a transition from a Tfh resting state to an activated one (Figure S4A). Moreover, addition of polarizing cytokines greatly enhanced the frequency of naive, memPD-1^neg^- and memPD-1^pos^-derived Tfh^D3^ cells as compared with the culture without cytokines (Figure S4B). Furthermore, IL-21 secretion and ICOS expression were potentiated for every Tfh^D3^ subset, independently of their origin (Figures S4C and S4D). These data support the idea that an antigen-stimulated lymphoid environment complemented with appropriate cytokines known to support Tfh cell development could favor acquisition of Tfh cell functions by any CD4^+^ T cell subtype.

To investigate whether orientation of each CD4^+^ T cell subset toward Tfh^D3^ was sustained by a specific transcriptional program, we used Venn diagram representations to highlight the overlap and specificities between sets of differentially expressed genes that are related to Tfh cell biology (Table 2). A core of multiple genes involved in Tfh cell biology overlapped between the transition from *ex vivo* CD4^+^ T cell subsets to their Tfh^D3^ cell relatives (Figure 3H). Molecules involved in Tfh cell signaling as STAT1 (Choi et al., 2013); in Tfh cell function as TNFRSF4 (OX40), SLAMF1, and CXCL13 (Crotty, 2019); and in the Tfh cell transcription program as IRF4 and ZBTB7 (encoding for Thpok) (Kwon et al., 2009; Vacchio et al., 2019) were upregulated during the transition from *ex vivo* CD4^+^ T cells to Tfh^D3^ cells. Molecules associated with Tfh cell regulation of PRDM1 and IL2RA (Ditoro et al., 2018; Johnston et al., 2009) were also found to be upregulated, suggesting that establishment of the Tfh cell program is concomitantly associated with expression of regulatory checkpoints. Additional Tfh cell transcription factors were exclusively upregulated in naive and memPD-1^neg^-derived Tfh^D3^ as MAF (Andris et al., 2017). Interestingly, Bcl6 was exclusively upregulated in memPD-1^neg^-derived Tfh^D3^. We found that transcription factors KLF2 and BACH2, identified as two inhibitors of the Tfh cell development (Choi et al., 2020; Lahmann et al., 2019; Lee et al., 2015), were downregulated in the transition from each *ex vivo* CD4^+^ T cell subsets to Tfh^D3^. Naive-derived Tfh^D3^ were associated with downregulation of PRKD2, which inhibits the transition from naive CD4^+^ T cells to Tfh (Misawa et al., 2020). CCR7 was downregulated in the transition from naive CD4^+^ T to Tfh^D3^, as previously reported (Haynes et al., 2007). Downregulation of STAT4, which is involved in Tfh/Th1 commitment, was shared between naive and memory-derived Tfh^D3^. In sum, transition of each CD4^+^ T cell subset toward Tfh^D3^ is characterized by its own “original” transcriptional program including regulation of genes implicated in Tfh cell differentiation as well as in T cell activation. Altogether, these data suggest that heterogeneous Tfh cell profiles observed *ex vivo* and *in vitro* at day 3 could be driven by the differentiation of multiple CD4^+^ T cell subsets, differing from each other by their maturation and their activation status.

**Table 2:** Selected genes involved in Tfh cell biology

Transcription factors			Functionality		Regulation	Positioning	Activation	Signaling
Tfh phenotype enhancers	Tfh phenotype repressors	Others	Cytokines/Chemokines	Co-stimulation				
BCL6	PRDM1	FoxP3	IL21	ICOS	IL1R1	CCR5	IL7R	STAT1
BATF	FOXO1	TBX21	CXCL13	CD28	IL2RA	CXCR4	MKI67	STAT3
IRF4	KLF2	RORC	IFNG	CD40LG	BTLA	CCR6	CD38	STAT4
MAF	PRKD2	GATA3	TNF	CTLA4	PDCD1	CCR7	CD44	STAT5A
ZBTB7B (Thpok)	BACH2		IL2	SH2D1A (SAP)	IL1R2	CXCR3	CD69	
TOX2	ID2		IL10	TNFRSF4 (Ox40)	IL6R	CXCR5		
ASB2			IL13	CD27	FAS	SELL (CD62L)		
TCF3 (E2A)			IL4	SLAMF1		S1PR1		
TCF7 (TCF-1)				TNFRSF18 (GITR)				
LEF1								

Based on the literature, we reviewed several molecules whose seminal role in Tfh cell development and function was shown.

### Tfh cell origins sustain Tfh cell heterogeneity at the peak of the antigenic stimulation

**Figure 4:**
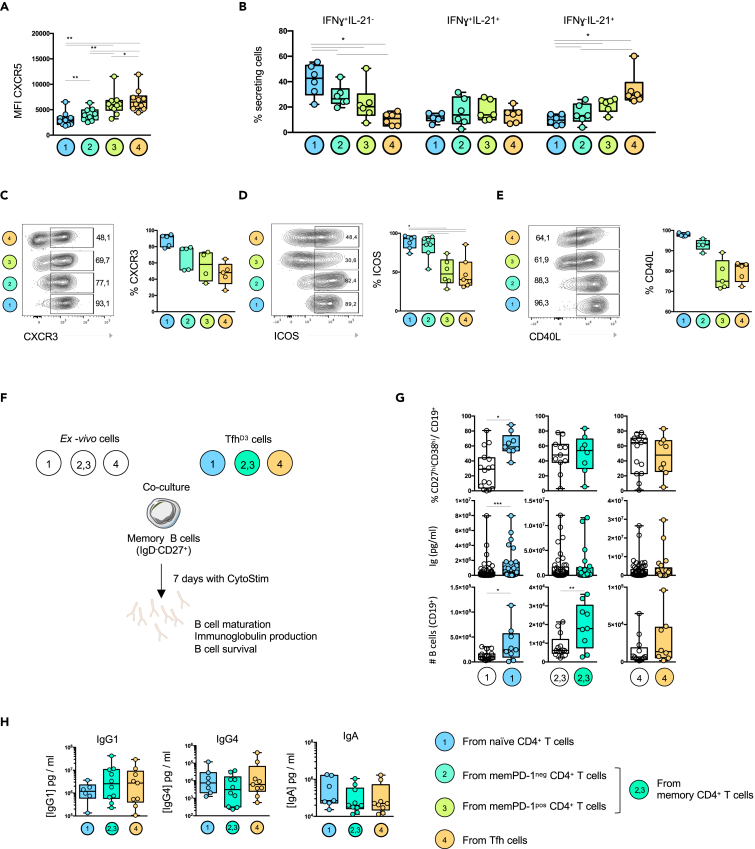
Distinct CD4^+^ T cell subsets contribute to the generation of Tfh with heterogeneous functional profiles (A) Mean fluorescence intensity of the CXCR5 marker expressed by CXCR5^+^ PD-1^+^ cells derived from (1) naive, (2) MemPD-1^neg^, (3) MemPD-1^neg^ and Tfh. (B) Frequency of IL-21- and/or IFNγ-positive cells among CXCR5^+^ PD-1^+^ cells at day 3. (C**–**E) Representative flow plots showing CXCR3, ICOS, and CD40L expression by CXCR5^+^ PD-1^+^ cells (left panel) and frequency of CXCR3-, ICOS-, and CD40L-positive cells among CXCR5^+^ PD-1^+^ cells at day 3 (right panel). (F) *Ex vivo* cells or their respective Tfh^D3^ counterparts obtained after 3 days of splenocyte culture were co-cultured with autologous B cells for 7 days. (G) Box plots represent the frequency of CD27^+^ CD38^+^ cells among CD19^+^ cells, the concentration of total immunoglobulins and the absolute number of live B cells after co-culture. (H) Quantification of IgG1, IgG4, and IgA in the co-culture supernatants. Each symbol (A–H) represents an individual donor. (A–H) A Wilcoxon matched pairs test was performed, ∗, p < 0.05; ∗∗, p < 0.005; ∗∗∗, p < 0.001.

To better characterize induced Tfh^D3^ of different origins, we first analyzed the intensity of CXCR5 expression, which mirrors Tfh cell maturation from non-GC to GC status (Kumar et al., 2021). The lowest CXCR5 mean fluorescence intensity was found in naive-derived Tfh^D3^ cells, and the highest in Tfh-derived Tfh^D3^ cells (Figure 4A). Regarding the expression of memory and naive markers at D3, these data are in accordance with the mass cytometry results, where disparity of CXCR5 expression was observed among global Tfh^D3^ (Figures 2B and S2A). To test whether the level of CXCR5 expression might relate to specific functional profiles, we analyzed IL-21 and IFNγ secretion. Naive-derived Tfh^D3^ were associated with higher IFNγ secretion, whereas Tfh-derived Tfh^D3^ were associated with abundant IL-21 secretion (Figure 4B). The cytokine secretion profile of memPD-1^pos^-derived Tfh^D3^ was closer to that of Tfh-derived Tfh^D3^, whereas memPD-1^neg^-derived Tfh^D3^ harbored a cytokine secretion profile closer to that of naive-derived Tfh^D3^. Coherently with a study showing that IFNγ secretion is related to CXCR3 expression in tonsillar Tfh (Brenna et al., 2020), we found that CXCR3 expression was higher in naive-derived Tfh^D3^ (Figure 4C). This higher expression of Th1 markers by naive-derived Tfh^D3^ might reflect the hybrid Tfh/Th1 profile, already described at an early stage of Tfh cell differentiation (Song and Craft, 2019). Regarding ICOS expression, naive and memPD-1^neg^-derived Tfh^D3^ were enriched in ICOS^+^ cells compared with memPD-1^pos^ and Tfh-derived Tfh^D3^ (Figure 4C). We next evaluated the expression of CD40L, which is essential to the function of Tfh (Crotty, 2019). The CD40L expression pattern followed that of ICOS, suggesting that naive and memPD-1^neg^-derived Tfh^D3^ provide more costimulatory signals than memPD-1^pos^ and Tfh-derived Tfh^D3^ (Figures 4D and 4E). Overall, our results suggest that the heterogeneous landscape of Tfh might result from the distinct contribution of naive and memory CD4^+^ T cells to the global Tfh^D3^ cell pool.

We next evaluated the functionality of Tfh^D3^ cells derived from each CD4^+^ T cell subset, focusing on B cell maturation, total immunoglobulin (Ig) production and B cell survival (Figure 4F). As compared with their native counterpart, naive-derived Tfh^D3^ cells exhibited an increased capacity to provide signals required for B cell maturation and survival. Coherently with the increased frequency of CD27^hi^CD38^hi^ plasma cells, Ig production was higher in co-culture with naive-derived Tfh^D3^ than with their native counterpart (Figure 4G). Tfh^D3^ derived from memPD-1^neg^ and memPD-1^pos^ CD4^+^ T cells, which were grouped because of the limited amount of available memory cells, were more efficient in helping B cell survival than their precursors, but did not promote higher B cell maturation and Ig production. Finally, Tfh-derived Tfh^D3^ showed similar capacities to provide B cell help as compared with Tfh^D0^ cells (Figure 4G). As CD4^+^ T cell subsets differ in their proliferative ability during co-culture, variation in B cell maturation could result from quantitative rather than qualitative interactions. To investigate this, we compared the numbers of CD4^+^ T cells present at the end of the co-culture with B cells (Figure S5). Although equivalent numbers of naive CD4^+^ T cells and naive-derived Tfh^D3^ were found at the end of the B cell co-cultures, the frequency of plasma cells was higher with naive-derived Tfh^D3^, showing that B cell maturation resulted more from qualitative help than from a higher frequency of CD4^+^ T cell partners (Figure S5). In this line, although Tfh^D0^ did not proliferate as much as naive CD4^+^ T cells, they induced more B cell maturation. Therefore, gain of B cell help functions varies according to the origin of Tfh.

As Tfh^D3^ harbored heterogeneous phenotypic profiles, we hypothesized that distinct Tfh^D3^ cell subsets could promote distinct B cell responses. Thus, we measured Ig subtypes in the co-culture supernatants. Although there was great variability between donors (n = 9) and we did not highlight any drastic B cell help specificities in the function of each Tfh^D3^ subset, some trends seemed to emerge. We found that Tfh and memory-derived Tfh^D3^ cells induced a slight increase of IgG1 production in comparison with naive-derived Tfh^D3^ (4- to 5-fold increase), whereas less production of IgG4 was obtained with memory-derived Tfh^D3^ (2.5- to 6-fold decrease). Conversely, naive-derived Tfh^D3^ were more associated with the promotion of IgA production (1.8- to 2.8-fold increase) (Figure 4H). Altogether, these data suggest that naive and memory CD4^+^ T cell subsets contribute to the pool of Tfh^D3^, resulting in the generation of multiple Tfh profiles, which in turn display slightly distinct B cell help properties.

### HIV(-1) infection shapes Tfh cell differentiation

**Figure 5:**
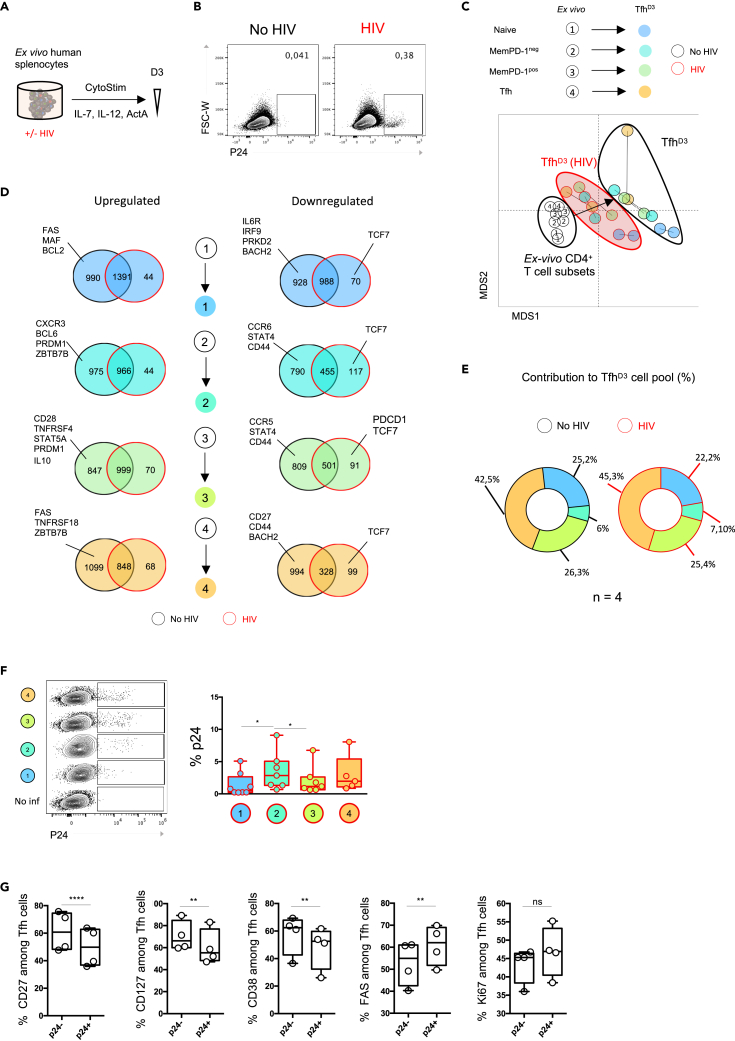
HIV infection shapes Tfh cell differentiation and functions (A) Splenocytes were stimulated according to the previously described protocol in the presence of HIV lab strain (Yu2b). (B) Representative flow plots of p24 staining among splenocytes after 3 days of culture with HIV or not. (C) RNA sequencing was performed on Tfh^D3^ cells derived from distinct CD4^+^ T cell subsets with and without HIV. Multidimensional scaling was performed to visually cluster different CD4^+^ T cell populations based on their transcriptional profile (8,593 genes). (D) RNA sequencing was performed on Tfh derived from each CD4^+^ T cell subset in the presence of HIV-1 infection or not. Differentially expressed genes were analyzed between Tfh and their original counterpart. Venn diagram representing (un)shared downregulated and upregulated genes. Genes specifically involved in Tfh cell biology were analyzed (referred to in Table 1). (E) Contribution of each CD4 T cell subset to total Tfh generated after 3 days of splenocyte culture (%). Data are plotted as the mean percentage contribution of each *ex vivo* CD4^+^ T cell subset: (1) naive CD4^+^ T cells, (2) (3) memPD-1^neg/pos^, and (4) Tfh to total Tfh^D3^ cells after splenocyte culture. (F) Representative flow plot of p24 staining in Tfh derived from distinct CD4^+^ T cell subsets. (G) Frequency of CD127-, CD27-, CD38-, FAS-, and ki67-positive cells among Tfh that are infected (p24^pos^) or not (p24^neg^). Each symbol (A–E) represents an individual donor. (E and G) A paired Student’s t test was performed, ∗p < 0.05, ∗∗p < 0.01. (F and G) A Wilcoxon matched pairs test was performed; ∗, p < 0.05; ∗∗, p < 0.005; ∗∗∗, p < 0.001.

In the context of HIV infection, we and others have shown an accumulation of dysfunctional Tfh in secondary lymphoid organs from chronically infected patients (Colineau et al., 2015; Cubas et al., 2013; Lindqvist et al., 2012; Perreau et al., 2013). Thus, we hypothesized that our culture system provides a good model to assess whether and how the multiple pathways of Tfh generation are modulated by HIV infection. Stimulated splenocytes were exposed to HIV infection using a CCR5-tropic HIV-1 strain (HIV_Yu2b_) (Figure 5A). First, we checked that our protocol led to HIV_Yu2b_ infection of splenocytes by analyzing p24 expression at day 3 after infection (Figure 5B). After HIV_Yu2b_ exposure splenocytes were infected, ranging from 0.15% to 2.97% of p24^+^ cells, which were not detected in the presence of reverse transcriptase inhibitors (not shown) and thus resulted from productive infection. We then isolated Tfh^D3^ generated under HIV_Yu2b_ infection and compared their transcriptome profile to that of uninfected controls (n = 2 donors). A multidimensional scaling representation of the transcriptome revealed that HIV_Yu2b_ exposure strongly impacts the genetic program driving Tfh cell differentiation. Tfh^D3^ generated under HIV_Yu2b_ infection showed an intermediate transcriptomic profile between *ex vivo* CD4^+^ T cell subsets and Tfh^D3^ uninfected controls (Figure 5C). A total of 990 genes were not upregulated in naive-derived Tfh^D3^ upon HIV_Yu2b_ infection compared with infection-free conditions (Figure 5D). An equivalent number of non-upregulated genes was found for other cell transitions. These genes were exclusive to each transition pathway, suggesting that HIV_Yu2b_ infection selectively impacts the transcriptional program depending on the Tfh^D3^ cell precursor. To evaluate more precisely whether HIV_Yu2b_ infection affected the Tfh cell differentiation program, we then focused on the expression of Tfh-related genes (Table 2). We found that Tcf7, which encodes Tcf1 and is involved in early induction of Bcl6 (Choi et al., 2015; Xu et al., 2015), was downregulated under HIV_Yu2b_ infection in all CD4^+^ T cell transitions toward the Tfh^D3^ profile (Figure 5D). During the transition from naive CD4^+^ T cells to Tfh^D3^ under HIV_Yu2b_ infection, we observed no downregulation of PRDK2 and BACH2, two inhibitors of the Tfh cell program. Coherently, no MAF upregulation was observed during the transition of naive CD4^+^ T cells toward Tfh^D3^, whereas this factor is implicated in early Tfh cell commitment after immunization (Andris et al., 2017). Similarly, we found no upregulation of Bcl6, PRDM1, and ZBTB7B (Thpok) in memPD-1^neg^-derived Tfh^D3^ cells under HIV_Yu2b_ infection. These results suggest that naive and memPD-1^neg^-derived Tfh^D3^ might harbor a defective Tfh cell phenotype. Indeed, CD28 and TNSFR4 (OX40) were not upregulated during the transition of memPD-1^pos^ cells toward Tfh^D3^ (Figure 5D), which is in accordance with defective co-stimulatory functions reported in splenic Tfh from chronically infected patients (Colineau et al., 2015). Hence, PDC1 was downregulated in memPD-1^pos^-derived Tfh^D3^ cells. Moreover, STAT5A and PRDM1, two key regulators of Tfh cell development, were not upregulated under HIV_Yu2b_ infection in memPD-1^pos^-derived Tfh^D3^. Overall, our transcriptional analysis showed that Tfh cell developmental and functional programs were altered by HIV_Yu2b_ infection and that the effect of HIV infection on the Tfh^D3^ cell subsets varied depending on their precursors. Surprisingly, cytometry analysis of Tfh^D3^ cell subsets did not indicate a major impact of HIV_Yu2b_ infection on global Tfh^D3^ cell proportion regarding their origin (Figure 5E), suggesting that HIV_Yu2b_ infection did not preferentially orient any CD4^+^ T cell subsets toward Tfh^D3^ at this time point. Coherently, HIV_Yu2b_ infection did not induce any preferential cell death among the Tfh^D3^ cell subsets (Figure S6).

Tfh are a major HIV reservoir compartment, which is one of the major obstacles to HIV eradication. Therefore, we investigated the infectious status of Tfh^D3^ subsets derived from various CD4^+^ T cell subsets. We found a preferential infection of memPD-1^neg^-derived Tfh^D3^ compared with memPD-1^pos^-derived Tfh^D3^ (Figure 5F), suggesting that memPD-1^neg^-derived Tfh^D3^ could preferentially contribute to the HIV reservoir. Finally, to evaluate whether HIV_Yu2b_ infection contributes to the altered phenotype of Tfh^D3^ cell subsets, we performed mass cytometry analysis to examine phenotypic differences between p24^neg^ and p24^pos^ Tfh^D3^ (Figure 5G). Interestingly, as compared with p24^neg^ Tfh^D3^, the percentage of p24^pos^ Tfh^D3^ displaying an activated phenotype (CD127, CD27, CD38) was reduced, whereas FAS was overexpressed (Figure 5G). The defective expression of activation markers by p24^pos^ Tfh^D3^ confirmed transcriptomic analysis (Figure 5D). Finally, a trend to more expression of ki67 was observed in p24^pos^Tfh^D3^. Thus, p24^pos^Tfh^D3^ presented a defective activation status while maintaining higher expansion ability.

Altogether, these results suggest that qualitative alterations observed in the Tfh cell compartment could result from the differential impact of HIV infection on the transition of *ex vivo* CD4^+^ T cell subsets toward Tfh^D3^ and from the capacity of Tfh^D3^ cells to sustain HIV reservoirs. These data suggest that many parameters, including the pathway of Tfh cell differentiation, could contribute to the accumulation of dysfunctional Tfh and the establishment of HIV reservoirs in lymphoid organs.

## Discussion

Most research investigating Tfh cell biology is based on the use of PBMCs, and few recent studies integrate the microenvironment of lymphoid organs for the study of Tfh cell responses. In these models, cell suspensions from lymphoid organs are mainly used to test the immunogenicity of vaccine candidates or drugs (Schmidt et al., 2020; Wagar et al., 2021). Among complementary approaches, lymphoid tissue explant models enable the study of the spread of HIV infection (Grivel and Margolis, 2009) in a situation much closer to *in vivo* conditions than our approach, although they do not allow the study of the impact of HIV infection on Tfh development. Therefore, we designed an alternative lymphoid cell-based model that makes a valuable contribution to the study of Tfh cell development and the impact of HIV infection on it. We took advantage of antigen-experienced splenocytes to promote functional Tfh including GC Tfh, which present similarities with those generated *in vivo* (Brenna et al., 2020; Vella et al., 2019). Moreover, these functional induced Tfh were susceptible to HIV infection.

Our data clearly showed that addition of cytokines known to support Tfh development and functions are strong potentiators of Tfh^D3^ induction and function regardless of their origin. These results are particularly coherent with recently published studies showing that adjuvanting HIV vaccine candidate with activin A promotes Tfh responses in a simian model (Carnathan et al., 2020) and that production of IL-12 and activin A by tonsillar myeloid cells sustains Tfh development (Durand et al., 2019). Considering that a single cytokine may vary in its effect depending on the micro-environment (Touzot et al., 2014), integration of cellular and molecular factors related to lymphoid organs is one strength of our culture system in comparison with co-culture assays.

One can assume that varying molecular and cellular environments might impact the induction of Tfh cell responses. Indeed, using our experimental design, antigen-experienced PBMCs did not lead to the generation of fully differentiated GC Tfh. Recently published data comparing Tfh isolated from tonsils and from blood showed that independent tissues present distinct proportions of Tfh in different maturation stages (Kumar et al., 2021). Here, our data provide new insights suggesting that the lymphoid environment is required to support the generation of fully differentiated GC Tfh. Indeed, many molecular or cellular factors would explain the propensity of splenocytes to support complete Tfh cell differentiation. In comparison with PBMCs, splenocytes are enriched in pro-Tfh subsets such as cDC2 cells and macrophages (Durand et al., 2019) and data not shown). Furthermore, compared with PBMCs, splenocytes are enriched in B cells, which are likely to play a role in the differentiation of Tfh in humans (Chavele et al., 2015).

We have shown that, in our model, Tfh cell induction requires T cell receptor signaling and polarizing cytokines. Thus, the lymphoid microenvironment initiates the Tfh differentiation program concomitantly with CD4^+^ T cell activation. Antigen-experienced splenocytes led to highly reproducible Tfh responses, which peaked 3 days after stimulation and then declined at day 5, indicating transitory T cell activation. Since Tfh are maintained with chronic exposure to antigen in lymphoid organs, one would expect that a second antigenic stimulation would maintain Tfh cell generation over time. However, in our culture system, the superantigen magnified CD4^+^ T cell activation and induced deep changes in the cellular composition of the splenocyte cell suspension, thus impeding Tfh cell program maintenance. Consequently, multiple antigenic stimulations would require further settings of our experimental design, such as renewal of lymphoid cells.

We have demonstrated that any subset of CD4^+^ T cells, including memory CD4^+^ T cells, can shift to a Tfh profile as early as day 3 after stimulation, leading a gradient of Tfh phenotype and functions. In our experimental conditions, we evidenced specific trajectories linked to the activation status of CD4 T cells (Figure 2). Previous *in vitro* studies have shown that memory CD4^+^ T cells can acquire Tfh features upon stimulation (Del Alcazar et al., 2019; Jacquemin et al., 2015; Lu et al., 2011; Pattarini et al., 2017). Here, we showed that orientation of memory CD4^+^ T cells toward the Tfh profile was sustained by a specific Tfh differentiation program. Indeed, analysis of the DEG between D0 and D3 suggested that each transition from *ex vivo* CD4^+^ T cell subsets to their Tfh^D3^ counterparts followed specific pathways of differentiation, even though a core of multiple genes involved in Tfh cell biology was conserved (Figure 3). Our experimental design induced Tfh activation into highly functional GC Tfh^D3^, whereas naive-derived Tfh^D3^ appeared less mature and memory-derived Tfh^D3^ cells presented an intermediate phenotype (Figures 2 and 3). This could be due to a delay in the acquisition of Tfh cell features by naive CD4^+^ T cells, which are supposed to require a multistep differentiation pathway (Crotty, 2014). Unfortunately, since the tracking of CD4^+^ T cells was not possible after 3 days of culture, we could not test the percentage and the phenotype of naive-derived Tfh after 5 and 10 days. Hence, high proportions of Tfh^D3^ would result from the differentiation of various CD4^+^ T cell subsets displaying intrinsic capacities to acquire Tfh cell features.

Furthermore, our data suggest that Tfh^D3^ derived from distinct CD4^+^ T cell origins provide different B cell help. For instance, even though naive-derived Tfh^D3^ produced less IL-21 than memory-derived Tfh^D3^, they expressed more ICOS and CD40L. We propose here that naive-derived Tfh^D3^ keep the expression of CD40L on their surface to interact with B cells and complete their differentiation into GC Tfh, whereas CD40L expression is downregulated on more mature Tfh^D3^ cells to possibly prevent the activation of non-cognate B cells (Yellin et al., 1994). CD127 expression appeared to reflect distinct stages of Tfh differentiation or activation (Figure 2D). Hence, expression of CD127 was lower in Tfh^D3^ than in their original counterpart. Coherently with the literature reporting low expression of CD127 on GC Tfh (Iyer et al., 2015; Lim and Kim, 2007), we identified a mature GC cluster as the one that expressed the least CD127 among Tfh^D3^ (Figure 2D).

However, such phenotypic heterogeneity does not translate into dramatic differences in the capacities of Tfh^D3^ to induce B cell maturation and Ig production (Figure 4). Of note, we performed T-B cell cocultures with memory B cells that are more prompt to mature than naive B cells (Locci et al., 2016; Pattarini et al., 2017; Ugolini et al., 2018). Consequently, memory B cells are probably less sensitive to the various B cell help abilities of distinct CD4^+^ T cells. In the same line, isotype class switching and maturation of antigen affinity would be of interest in evaluating the activation of Tfh^D0^ into GC Tfh^D3^.

The capacity of memPD-1^pos^ CD4^+^ T cells to generate Tfh was higher in comparison with memPD-1^neg^ CD4^+^ T cells, showing that Tfh cell generation differs according to T cell activation status. Lymphoid tissues are particularly enriched in memPD-1^pos^ CD4^+^ T cells as compared with PBMCs. The propensity of memPD-1^pos^ CD4^+^ T cells to orient toward a Tfh cell profile might confer a significant advantage by rapidly sustaining and diversifying B cell responses after antigenic exposure. However, rapid Tfh cell conversion could be deleterious in HIV infection where memPD-1^pos^ CD4^+^ T cells accumulate (Del Alcazar et al., 2019) and lymphoid structures are altered (Estes, 2013). Thus, unregulated Tfh cell generation could be induced in this context.

Transcriptomic analysis revealed that HIV infection deeply impacts the transcriptomic program of Tfh cell differentiation. Moreover, deep immunophenotyping evidenced defective activation of p24^pos^ Tfh^D3^. Under *in vitro* HIV infection, we showed that the percentage of CD127^+^ cells among p24^pos^ Tfh^D3^ is reduced compared with p24^neg^ Tfh^D3^ (Figure 5D). This observation is in accordance with previous work showing that expression of CD127 is lost on a large proportion of peripheral T cells in HIV-1-infected patients with lymphopenia (Chiodi et al., 2017). One can suppose that CD127 loss contributes to the higher susceptibility of p24^pos^ Tfh^D3^ to cell death, which is coherent with the higher percentage of FAS^+^ cells among p24^pos^ Tfh^D3^ cells as compared with p24^neg^ Tfh^D3^. These results give new insight into the induction of defective Tfh in HIV-infected patients. Interestingly, we found a preferential infection of memPD-1^neg^-derived Tfh^D3^ compared with memPD-1^pos^-derived Tfh^D3^. However, as compared with uninfected control, this preferential infection does not result in a differential contribution of the distinct CD4^+^ T cell precursors to the overall Tfh^D3^ cell pool. Longer tracking of CD4^+^ T cell subsets would be helpful to investigate potential variations in the respective contribution of derived Tfh^D3^ subsets at later time points. Lastly, characterization of Tfh cell pathways focusing on HIV-specific CD4^+^ T cells would be very interesting. However, the length of *in vitro* culture is too short to expect the CD4^+^ T cell priming allowing the study of HIV-specific Tfh. Hence, using splenocytes from HIV-infected patients could be relevant for this purpose (Colineau et al., 2015).

Altogether, our experimental model provides first-order information on the multiple pathways of Tfh development and activation, which so far are unidentified in a human lymphoid environment. The applicability of this model to HIV infection allowed us to confirm functional defects of Tfh in HIV-infected patients in the light of the newly identified pathways of Tfh cell induction.

### Limitations of the study

This study mostly relies on the *in vitro* culture systems. Further studies are required to evaluate if the differences observed for naive- or memory-derived Tfh cells differentiated *in vitro* recapitulate the biology of Tfh cells *in vivo*.

## STAR★Methods

### Key resources table

**Table:** 

REAGENT or RESOURCE	SOURCE	IDENTIFIER
**Antibodies**		

PE-Vio770 anti-human CXCR5	Miltenyi Biotec	Cat# 130-117-358; RRID:AB_2733205
PE anti-human PD-1	Miltenyi Biotec	Cat# 130-117-384; RRID:AB_2727929
Percp-Vio770 anti-human CD3	Miltenyi Biotec	Cat# 130-113-141; RRID:AB_2725969
APC anti-human CD19	Miltenyi Biotec	Cat# 130-110-250; RRID:AB_2655809
Vioblue anti-human ICOS	Miltenyi Biotec	Cat# 130-100-737; RRID:AB_2656918
PE-Vio770 anti-human CD38	Miltenyi Biotec	Cat# 130-099-151; RRID:AB_2660384
Vioblue anti-human CD40L	Miltenyi Biotec	Cat# 130-109-470; RRID:AB_2655266
APC-Vio770 anti-human CD4	Miltenyi Biotec	Cat# 130-113-223; RRID:AB_2726034
FITC anti-human IFNγ	Miltenyi Biotec	Cat# 130-113-497; RRID:AB_2733587
APC anti-human IL-21	Miltenyi Biotec	Cat# 130-117-421; RRID:AB_2727941
PE-Vio615 anti-human CD45RA	Miltenyi Biotec	Cat# 130-118-789; RRID:AB_2732979
PE anti-human CD27	Miltenyi Biotec	Cat# 130-113-640; RRID:AB_2726194
APC anti-human Bcl6	BD Bioscience	Cat# 561525; RRID:AB_10898007
Vio Bright FITC anti-human CXCR3	Miltenyi Biotec	Cat# 130-118-673; RRID:AB_2734057
FITC KC57 (anti-p24)	Beckman Coulter	Cat# 6604665; RRID:AB_1575987
Pure anti-human CCR5	Miltenyi Biotec	Cat# 130-122-313; RRID:AB_2801894
Pure anti-human CD56	Miltenyi Biotec	Cat# 130-108-016; RRID:AB_2658728
Pure anti-human CCR7	Miltenyi Biotec	Cat# 130-122-300; RRID:AB_2801881
Pure anti-human CD62L (Custom Reagent Cell Analysis)	Miltenyi Biotec	Cat# 130-113-625; RRID:AB_2733829
Pure anti-human IL-1R2 (Custom Reagent Cell Analysis)	Miltenyi Biotec	Cat# 130-126-459
Purified anti-human IL-1R1 Polyclonal Ab	Bio-techne	Cat# AF269; RRID:AB_355286
Purified anti-human BTLA Polyclonal Ab	Bio-techne	Cat# AF3354; RRID:AB_2065766
Anti-Human Ki-67 (B56)- 172Yb	Fluidigm	Cat# 3172024B; RRID:AB_2858243
Anti-Human CD152/CTLA-4 (14D3)-170Er	Fluidigm	Cat# 3170005B; RRID:AB_2858238
Anti-Human CD28 (CD28.2)-160Gd	Fluidigm	Cat# 3160003B; RRID:AB_2868400
Anti-Human CD134/OX40 (ACT35)-158Gd	Fluidigm	Cat# 3158012B
Anti-CD278/ICOS (C398.4A)-151Eu	Fluidigm	Cat# 3151020B
Anti-Human CD154/CD40L (24-31)-168Er	Fluidigm	Cat# 3168006B
Anti-Human CD196/CCR6 (11A9)-141Pr	Fluidigm	Cat# 3141014A
Anti-Human CD183/CXCR3 (G025H7)-156Gd	Fluidigm	Cat# 3156004B; RRID:AB_2687646
Anti-Human CD279/PD-1 (EH12.2H7)-155Gd	Fluidigm	Cat# 3155009B; RRID:AB_2811087
Anti-Human CD185/CXCR5 (RF8B2)-171Yb	Fluidigm	Cat# 3171014B; RRID:AB_2858239
Anti-Human HLA-DR (L243)-173Yb	Fluidigm	Cat# 3173005B; RRID:AB_2810248
Anti-Human CD38 (HIT2)- 144Nd	Fluidigm	Cat# 3144014B; RRID:AB_2687640
Anti-Human CD127/IL-7Ra (A019D5)-165Ho	Fluidigm	Cat# 3165008B; RRID:AB_2868401
Anti-Human CD27 (L128)- 162Dy	Fluidigm	Cat# 3162009B; RRID:AB_2756422
Anti-Human CD95/Fas (DX2)-152Sm	Fluidigm	Cat# 3152017B
Anti-Human CD8 (RPA-T8)- 146Nd	Fluidigm	Cat# 3146001; RRID:AB_2687641
Anti-Human CD19 (HIB19)- 142Nd	Fluidigm	Cat# 3142001; RRID:AB_2651155
Anti-Human CD4 (RPA-T4)- 145Nd	Fluidigm	Cat# 3145001; RRID:AB_2661789
Anti-Human CD45RO (UCHL1)-164Dy	Fluidigm	Cat# 3164007B; RRID:AB_2811092
Anti-Human CD45 (HI30)- Y89	Fluidigm	Cat# 3089003; RRID:AB_2661851
Anti-Human CD45RA (HI100)-143Nd	Fluidigm	Cat# 3143006B; RRID:AB_2651156
Anti-Human CD25 (2A3)- 149Sm	Fluidigm	Cat# 3149010B; RRID:AB_2756416
Anti-Human FoxP3 (259D/ C7)-159Tb	Fluidigm	Cat# 3159028A; RRID:AB_2811088
Anti-Human CD184/CXCR4 (12G5)-175Lu	Fluidigm	Cat# 3175001B

**Bacterial and virus strains**		

HIV-1 R5 strain YU2b	Cardinaud et al. (2017)	N/A

**Biological samples**		

PBMC	Etablissement français du sang	N/A
Spleens	Biomedecine Agency	N/A

**Chemicals, peptides, and recombinant proteins**		

Recombinant human Activin A, premium grade	Miltenyi	Cat# 130-115-010
Recombinant human IL-7, premium grade	Miltenyi	Cat# 130-095-363
Recombinant IL-12, premium grade	Miltenyi	Cat# 130-096-798
CytoStim™, human	Miltenyi	Cat# 130-092-173
Cell-IDTM Intercalator-Rh	Fluidigm	Cat# 201103B
Cell-IDTM Cisplatin-198Pt	Fluidigm	Cat# 201198
Cell-IDTM Intercalator-Ir	Fluidigm	Cat# 201192B
EQ Four Element Calibration Beads	Fluidigm	Cat# 201078
LIVE/DEAD™ Fixable Aqua	Thermofisher	Cat# L34957
Fixable Viability Dye eFluor™ 780	Thermofisher	Cat# 65-0865-14
Cell Trace Violet	Thermofisher	Cat# C34557
PMA	Sigma-Aldrich	Cat# P1585
Ionomycin	Sigma-Aldrich	Cat# I9657
Brefeldin A	Sigma-Aldrich	Cat# B6542
DNase	Sigma-Aldrich	Cat# 10104159001
Fc Block	Miltenyi	Cat# 130-059-901

**Critical commercial assays**		

BD Cytofix/Cytoperrm Kit	BD Biosciences	Cat# 554714
Transcription Factor Buffer Set	BD Biosciences	Cat# 562574
Maxpar® X8 Antibody Labeling Kit, 150Nd—4 Rxn	Fluidigm	Cat# 201150A
Maxpar® X8 Antibody Labeling Kit, 174Yb—4 Rxn	Fluidigm	Cat# 201174A
Maxpar® X8 Antibody Labeling Kit, 148Nd—4 Rxn	Fluidigm	Cat# 201148A
Maxpar® X8 Antibody Labeling Kit, 153Eu—4 Rxn	Fluidigm	Cat# 201153A
Maxpar® X8 Antibody Labeling Kit, 169Tm—4 Rxn	Fluidigm	Cat# 201169A
Maxpar® X8 Antibody Labeling Kit, 166Er—4 Rxn	Fluidigm	Cat# 201166A
Maxpar® X8 Antibody Labeling Kit, 154Sm—4 Rxn	Fluidigm	Cat# 201154A
Maxpar® X8 Antibody Labeling Kit, 176Yb—4 Rxn	Fluidigm	Cat# 201176A
Maxpar® X8 Antibody Labeling Kit, 147Sm—4 Rxn	Fluidigm	Cat# 201147A
Calcium Phosphate Transfection Kit	Sigma-Aldrich	Cat# CAPHOS-1KT
RNeasy Mini Kit	Qiagen	Cat# 74106
Cytokine 7-Plex Human ProcartaPlex™ Panel 1C	Thermo Fisher Scientific	Cat# EPX070-10010-901; RRID:AB_2576087

**Experimental models: Cell lines**		

293T	ATCC	Cat# CRL-3216

**Software and algorithms**		

BD FACSDiva Software	BD Biosciences	http://www.bdbiosciences.com/instruments/software/facsdiva/index.jsp BD FACSDiva Software (RRID:SCR_001456)
FlowJo v10	FlowJo LLC, USA	FlowJo (RRID:SCR_008520)
GraphPad Prism 6	GraphPad	http://www.graphpad.com/GraphPad Prism (RRID:SCR_002798)
R software	R software	http://www.r-project.org/R Project for Statistical Computing (RRID:SCR_001905)
UMAP algorithm	McInnes et al., 2018	https://github.com/lmcinnes/umap Umap (RRID:SCR_018217)
“CytoTree” R package	Dai et al. (2021)	https://github.com/JhuangLab/CytoTree
Cutadapt	Martin (2011)	http://code.google.com/p/cutadapt/cutadapt (RRID:SCR_011841)
Salmon	Patro et al. (2017)	https://combine-lab.github.io/salmon/Salmon (RRID:SCR_017036)
DESeq2	Love et al. (2014)	https://bioconductor.org/packages/release/bioc/html/DESeq2.html DESeq2 (RRID:SCR_015687)
EnrichR	Chen et al. (2013)	http://amp.pharm.mssm.edu/Enrichr/Enrichr (RRID:SCR_001575)

**Deposited data**		

https://doi.org/10.17632/zs4y45ctdw.1		

### Resource availability

#### Lead contact

Further information and requests for resources and reagents should be directed to and will be fulfilled by the lead contact, Stéphanie Graff-Dubois (stephanie.graff-dubois@sorbonne-universite.fr).

#### Materials availability

This study did not generate new unique reagents

### Experimental model and subject details

#### Patients

Spleens were obtained from healthy donors (n = 14). Informed consent and protocols were approved by the Biomedicine Agency. Fresh whole blood samples from healthy donors were obtained from the Etablissement Français du Sang. All spleen samples included in the study were collected following national ethical guidelines regulating the use of human tissues.

### Method details

#### Spleen processing and freezing

During delivery, splenic tissues were maintained at 15–22°C and RPMI 1640 (Thermo Fisher Scientific) supplemented with penicillin-streptomycin (100 U/mL, Thermo Fisher Scientific) was used as delivery medium. Spleens were comminuted mechanically in a culture dish containing RPMI with penicillin-streptomycin (100 U/mL, Thermo Fisher Scientific), filtered through a cell strainer (70 μm, Sigma Aldrich). After decantation, the cell suspension was transferred into a 50 mL tube (Falcon) containing Pancoll (Pan Biotech) and centrifuged. Then, cells were washed twice with RPMI medium and frozen at −150°C in medium containing fetal bovine serum (FBS) (Sigma Aldrich) with 10% dimethyl sulfoxide (DMSO) (Sigma Aldrich).

#### Splenocyte cultures

Cells were thawed and washed twice in pre-warmed RPMI and then resuspended in RPMI supplemented with 10% FBS, L-glutamine 2 mM, penicillin 2 units/mL, 1 mg/mL streptomycin (complete RPMI) and DNase (10 ng/mL, Sigma Aldrich) overnight. Cells were washed and stimulated for 3 hours with CytoStim (Miltenyi, 2 µL/million cells) in complete RPMI at 37°C. CytoStim is a bi-specific antibody which binds simultaneously to the TCR and to MHC, thus cross-linking effector and memory CD4 or CD8 T cells and antigen presenting cells. CytoStim provides a strong polyclonal T cell stimulation, without any TCR Vbeta restrictions. After centrifugation, cells were resuspended in a polarizing medium consisting of complete RPMI supplemented with 100 ng/mL activin A, 5 ng/mL IL-12, 4 ng/mL IL-7 (Miltenyi, premium grade). Splenocytes were cultured in 24-well plates (Dutscher) at the concentration of 2 million cells/1 mL/well for 3 days. For the cell tracking experiment, CD4^+^ T cell subsets of interest were sorted, using a BD FACSARIA II (BD Biosciences). Then, CD4^+^ T cells were stained with cell trace violet (Thermo Fisher) for 20 min at 37°C and mixed back into the conserved fraction containing all other cells. The same protocol as before was applied.

#### Assessment of B cell help by T-B co-culture

CD4^+^ T cells of interest were isolated from *ex vivo* or stimulated splenocytes using a BD FACSAria II (BD Biosciences), with over 95% purity. Fresh autologous total or memory B cells were sorted (CD19^+^CD27^+^IgD^−^). 20,000 CD4^+^ T cells and 20,000 B cells were co-cultured for seven days in the presence of CytoStim. The culture medium contained ½ medium from previous splenocyte stimulation and ½ complete RPMI. After seven days, maturation of B cells (CD27 and CD38) was assessed by flow cytometry and immunoglobulin concentrations were determined using Luminex with the antibody Isotyping 7-Plex Human ProcartaPlex^TM^ Panel (Thermo Fisher Scientific).

#### HIV production

Replicative HIV_Yu2b_ lab strain (CCR5 tropic) was generated as previously described (Cardinaud et al., 2017) by transfection of 293T cells with the Calcium Phosphate Transfection Kit (Sigma Aldrich). After transfection, cells were cultured in DMEM (Thermo Fisher Scientific) supplemented with 10% FBS, 2 mM L-glutamine, 2 units/mL penicillin, 1 mg/mL streptomycin. Supernatants containing HIV_Yu2b_ were harvested 3 times every 12 hours from transfection. After centrifugation for removal of cellular debris, supernatants were filtered and then frozen at −80°C. The Gag-p24 content of all viral stocks was measured using an ELISA (PerkinElmer).

#### Splenocyte infection by HIV

After CytoStim stimulation for 3 hours, splenocytes were exposed to HIV_Yu2b_ at a concentration ranging from 150 to 700 ng/mL of p24 for 4 million splenocytes. Infection was performed at 37°C for 3 hours in the presence of diethylaminomethyl (DEAE)–dextran (Sigma Aldrich) at 4 μg/mL. Then, splenocytes were washed twice and cultured for 3 days in a polarizing medium as before.

#### Flow cytometry staining and cell sorting

Before flow cytometry and cell sorting, splenocytes were systematically stained for cell viability using Viobility Dye (Miltenyi) at room temperature for 15 minutes. After two wash steps, splenocytes were stained in PBS 1 × 5% FBS with antibodies directed against chemokine receptor (CXCR5; CXCR3) (Miltenyi) for 10 minutes at 4°C. Splenocytes were washed and cell surface staining was performed (CD4, CD19, PD-1, ICOS) (Miltenyi). For detection of transcription factors Bcl6 and Foxp3, a Fixation/Permeabilization Kit (BD, Biosciences) was used according to the manufacturer’s instructions. For intra-cellular cytokine detection, cells were stimulated for 6 h at 37°C with phorbo 12-myristate 13-acetate (PMA, Sigma Aldrich 1 μg/mL) and ionomycin (Sigma Aldrich, 1 μg/mL), and brefeldin A was added after 1 h of incubation (BFA, 5 μg/mL). Cells were fixed with the Cytofix/Cytoperm kit (BD Biosciences) for detection of cytokines (IL-21, IFNγ and CD40L)(Miltenyi). Cells were analyzed on an LSRFortessa Cell Analyzer (BD Biosciences) or a CytoFLEX (Beckman Coulter). For cell sorting, cells were sorted using a BD FACSAria II (BD Biosciences). Prior to cell sorting, staining buffer (PBS 1 × 5% FBS) was supplemented with DNase (Sigma Aldrich) and EDTA (Thermo Fisher Scientific).

#### Mass cytometry profiling

Metal-conjugated antibodies are listed in Table 1. Three million cells per sample were stained. Cell viability was assessed by Cisplatin Cell-ID^TM^ (Fluidigm). Cells were washed with RPMI. Fc Block (Miltenyi) was diluted in a staining buffer (PBS 1X, 5% FBS) and added to avoid nonspecific staining, for 15 minutes at RT. After washing the cells, anti-chemokine antibodies (CXCR5, CXCR3, CXCR4, CCR5, CCR6, CCR7) were added in PBS 1 × 5% FBS at room temperature for 15 minutes. Other membrane markers were added next (CD45, CD56, CD19, CD11b, CD8, CD4, CD45RA, CD62L, CD45RO, Tim-3, PD-1, CD25, Fas, HLADR ICOS, Ox40, CD28, BTLA, CD57, CD127, CD27, IL-1R1, IL-1R2) and splenocytes were incubated for 30 additional minutes at room temperature. After washing, splenocytes were fixed using PBS 1X containing 2% paraformaldehyde (PFA) (Thermo Fisher Scientific) for 15 minutes at room temperature. After washing in a staining buffer, cells were resuspended in a residual volume and incubated for 10 minutes on ice. One mL of −20°C methanol (Sigma Aldrich) was added for 10 minutes on ice. After washing, splenocytes were stained for intracellular markers (Foxp3, SH2D1a/SAP, CD40L, Ki67, CTLA4) for 60 minutes at room temperature. Then, splenocytes were washed and cellular DNA was stained with Cell-ID-Intercalator-Ir-125 μM (Fluidigm) diluted in PBS 1 × 2% PFA for 24 hours at 4°C. Samples were next frozen at −80°C and thawed before acquisition with Element EQ beads on Helios (Fluidigm) at the cytometry core facility of Pitié-Salpêtrière Hospital.

Mass cytometry profiles were represented using the UMAP algorithm (McInnes et al., 2018) from “uwot” R package and the plot builder “ggplot” R package. Cell clusters were identified using a k-means algorithm (k = 8) from the “stats” R package, and were represented using the “ComplexHeatmap” R package.

#### Trajectory inference and pseudotime analysis

All steps of the analysis were performed using the “CytoTree” R package (Dai et al., 2021). Exported mass cytometry FCS data from total CD4^+^ T cells were imported, transformed with *cytofAsinh* method for 30 selected markers, scaled to range (0,1) and downsampled to the minimum number of cells from all FCS files. The preprocessed data and metadata (i.e. donor id and stage) were merged in a *CYT* object to perform clustering with *SOM* method and dimensionality reduction. Cell trajectory was inferred using the Minimum Spanning Tree (MST) method, and pseudotime calculation was conducted by defining root clusters as the ones containing naive cells (6, 8, 10 at D0; 6, 9, 13 at D3), following CD45RA/RO expression. Through tree plot and heatmap visualization, clusters were assigned to metaclusters, which identification was made by marker expression comparison.

#### Transcriptomic profiling

Total RNA was purified using the RNeasy Mini Kit (Qiagen). RNA integrity was assessed using the TapeStation System (Agilent) and all RIN of analyzed samples were greater than 8. RNA was sequenced using Illumina Novaseq (Illumina, 80 million reads per sample, read length of 100 base pairs). Sequenced reads were trimmed for quality using Cutadapt (Martin, 2011) and aligned using Salmon (Patro et al., 2017) on the Ensembl reference of the human transcriptome (version GRCh38). Gene expressions were analyzed using DESeq2 (Love et al., 2014). Functional enrichment analyses were performed using EnrichR (Chen et al., 2013).

### Quantification and statistical analysis

Differences were evaluated using Wilcoxon matched pairs test and paired Student’s t-test using GraphPad Prism 6 (GraphPad Software, La Jolla, CA, USA). p values are presented directly in the figures as follows: ns, p > .05 (not significant); ∗, p < .05, ∗∗; p < .005, ∗∗∗; p < .001.

## Data Availability

Raw data from Figures 2 and S2 were deposited on Mendeley at https://doi.org/10.17632/zs4y45ctdw.1.
