# Supplementary material for: Naive and memory CD4+ T cell subsets can contribute to the generation of human Tfh cells
Source: iScience. 2021 Dec 3;25(1):103566. doi: 10.1016/j.isci.2021.103566 (PMC8693005; doi:10.1016/j.isci.2021.103566)
Supplement: Document S1. Figures S1–S6 [file mmc1.pdf]

iScience, Volume *25*

Supplemental information

Naive and memory $CD4^+$ T cell subsets can contribute to the generation of human Tfh cells

Raphaël Jeger-Madiot, Romain Vaineau, Maud Heredia, Nicolas Tchitchek, Lisa Bertrand, Mathias Pereira, Océane Konza, Bruno Gouritin, Bénédicte Hoareau-Coudert, Aurélien Corneau, Catherine Blanc, Eric Savier, Pierre Buffet, Adrien Six, David Klatzmann, Arnaud Moris, and Stéphanie Graff-Dubois

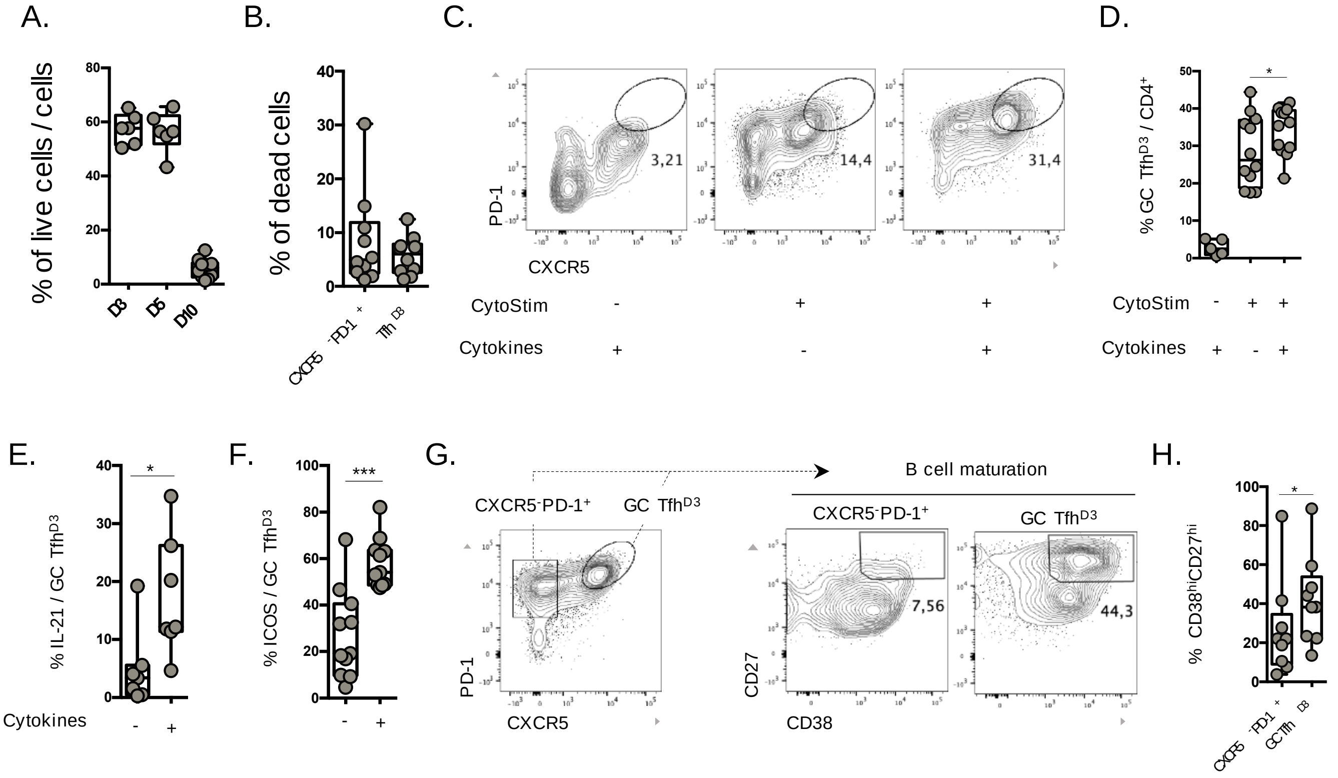

Figure S1. Lymphoid environment in polarizing conditions potentiates functional Tfh cell response, related to figure 1. (A) Percentage of live cells among CD4$^+$ T cells assessed by flow cytometry during the culture at day 3, 5 and 10. **(B)** Percentage of dead cells among non-Tfh (defined as CXCR5$^-$ PD-1$^+$) and Tfh^{D3} cells at day 3. **(C)** Representative flow plots showing CXCR5 and PD-1 expression on CD4$^+$ T cell splenocytes unstimulated or stimulated with CytoStim and then cultured in the presence or not of polarizing medium (IL-7, IL-12, activin A) for 3 days. (n = 5 – 12). **(D)** Percentage of GC Tfh^{D3} cells among CD4$^+$ T cells in conditions described in (B). **(E-F)** Frequency of IL-21-secreting cells (E) or ICOS$^+$ cells (F) among GC Tfh^{D3} cells generated in the presence or absence of polarizing cytokines (n = 9-12). **(G)** After 3 days of antigenic stimulation, GC Tfh^{D3} and CXCR5- PD-1+ CD4+ T cells were sorted and co-cultured with autologous CD19$^+$ B cells for 7 days. Representative flow plots showing CD27 and CD38 expression on CD19$^+$ cells after 7 days of culture. **(H)** Percentage of CD27- and CD38-positive cells among CD19$^+$ cells (n = 9). Each symbol represents an individual sample from an independent donor. A Wilcoxon matched pairs test was performed, *$p<0.05$, **$p<0.005$, ***$p <0.001$.

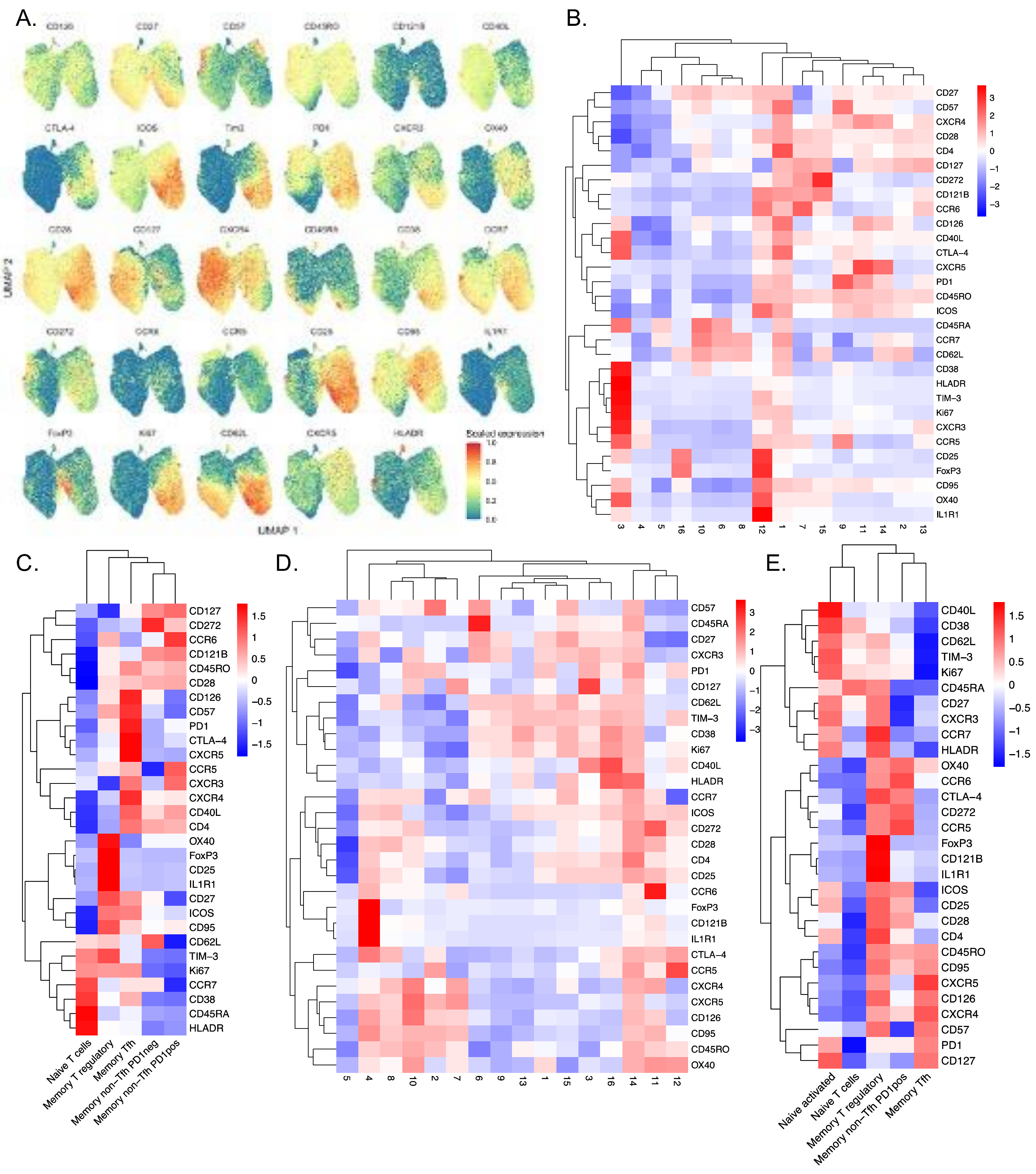

Figure S2. Memory Tfh are distinguished from other CD4^{+} T cell subsets by clustering on the same core markers at D0 and D3, related to figure 2. (A) Scaled expression of 29 markers by CD4^{+} CXCR5^{+} T cells analyzed by mass cytometry at D0 and D3. UMAP plots show the intensity of expression for selected markers at the single cell level. **(B and C)** Heatmap representations displaying clusters (B) and metaclusters (C) among total D0 CD4+ T cells, following the expression of 30 selected mass cytometry markers. **(D and E)** Heatmap representations displaying clusters (D) and metaclusters (E) among total D3 CD4+ T cells, following the expression of 30 selected mass cytometry markers.

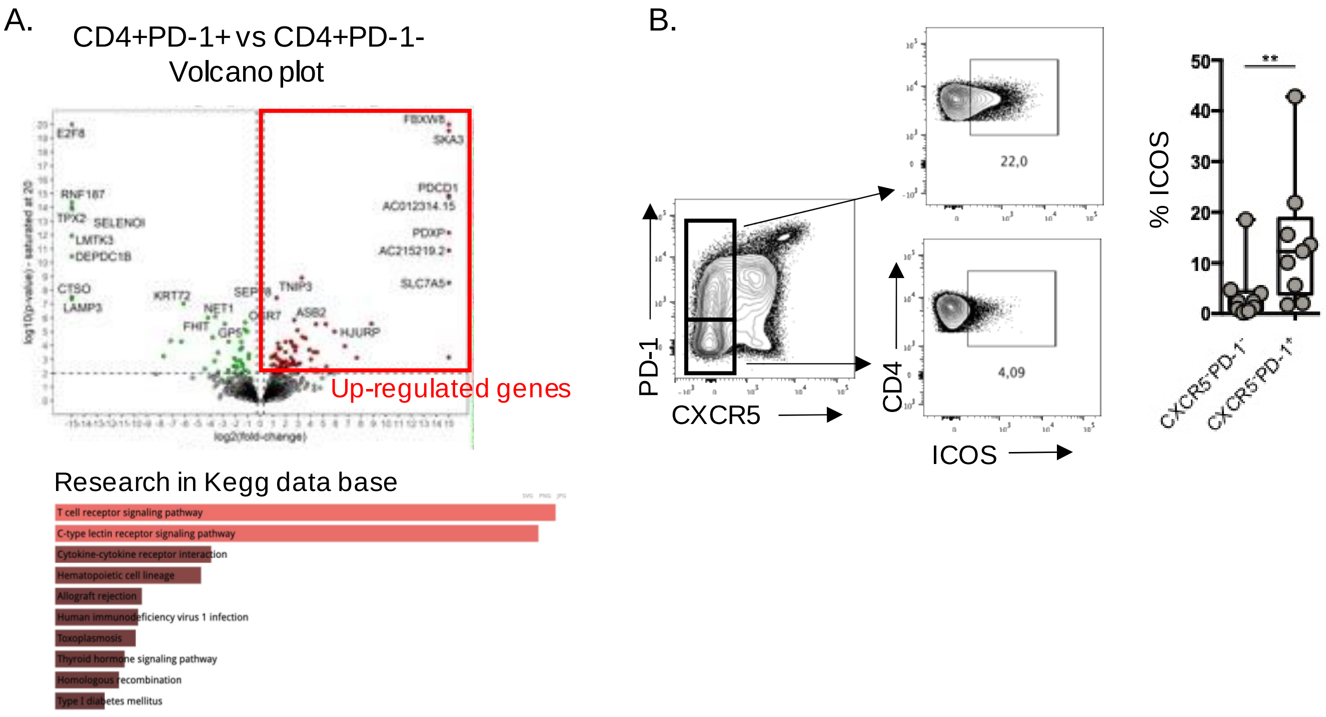

Figure S3. ***Ex vivo*** **memory CD4$^+$ PD-1$^+$ T cells display an activated profile in comparison to *ex vivo* memory CD4$^+$ PD-1$^-$ T cells, related to figure 3. (A)** CD4$^+$ PD-1$^+$ and PD-1$^-$ non-Tfh of two donors were sorted (n=2 donors) by flow cytometry after overnight resting of splenocytes. After RNAseq extraction, DEGs between the two populations were identified, the upregulated genes were selected and associated signatures were assessed using the KEGG database. **(B)** Representative flow plots showing the gating strategy for the identification of ICOS$^+$ cells and frequency of Tfh among non-Tfh CD4$^+$ expressing PD-1 or no (n=9). A Wilcoxon matched paired test was performed, *p<0.05, **p<0.005, ***p <0.001.

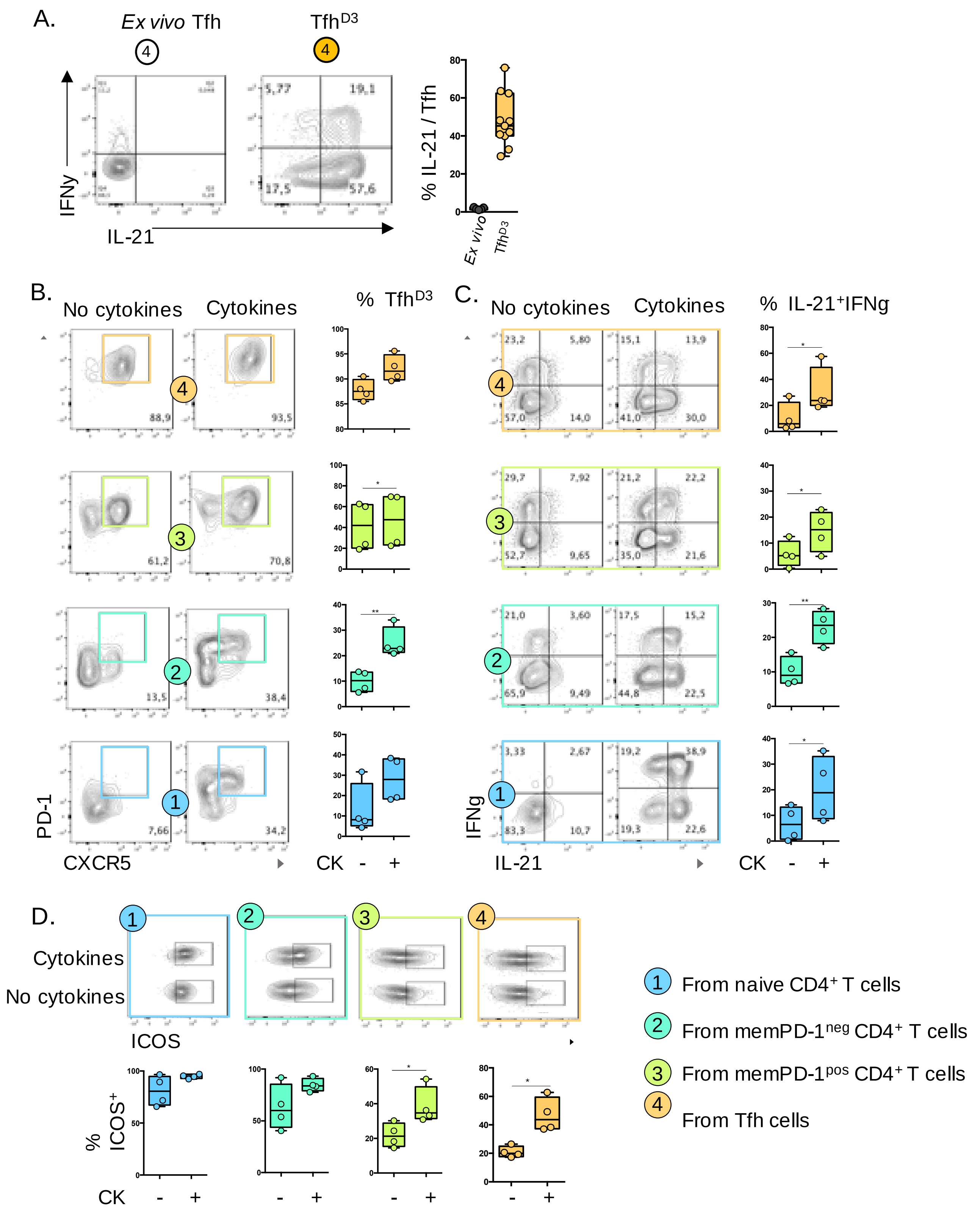

Figure S4. Activation and functionalization of *ex vivo* Tfh and induced Tfh after splenocyte stimulation in polarizing medium, related to figure 4. (A) Representative flow plots showing the gating strategy for the identification of IL-21$^+$ and IFNγ^+ cells and the frequency of IL-21 and IFNγ among *ex vivo* Tfh and activated Tfh^{D3} cells during the culture of stimulated splenocytes (n = 4 for *ex vivo*, n= 10 for Tfh^{D3}). **(B)** Representative flow plots showing the gating strategy for the identification of Tfh^{D3} after splenocyte stimulation with the presence or absence of polarizing cytokines (right) and the associated percentage among CD4$^+$ T cells (left) (n = 4). **(C and D)** Representative flow plots showing the gating strategy for the identification of IL-21$^+$,IFNγ^+ cells (C) and ICOS$^+$ cells (D) among Tfh^{D3} cells after splenocyte stimulation with the presence or not of polarizing cytokines (right) and the associated percentage among Tfh^{D3} cells (left) (n = 4). A paired Student's t-test was performed in C, $^*p<0.05$, $^{**}p<0.01$.

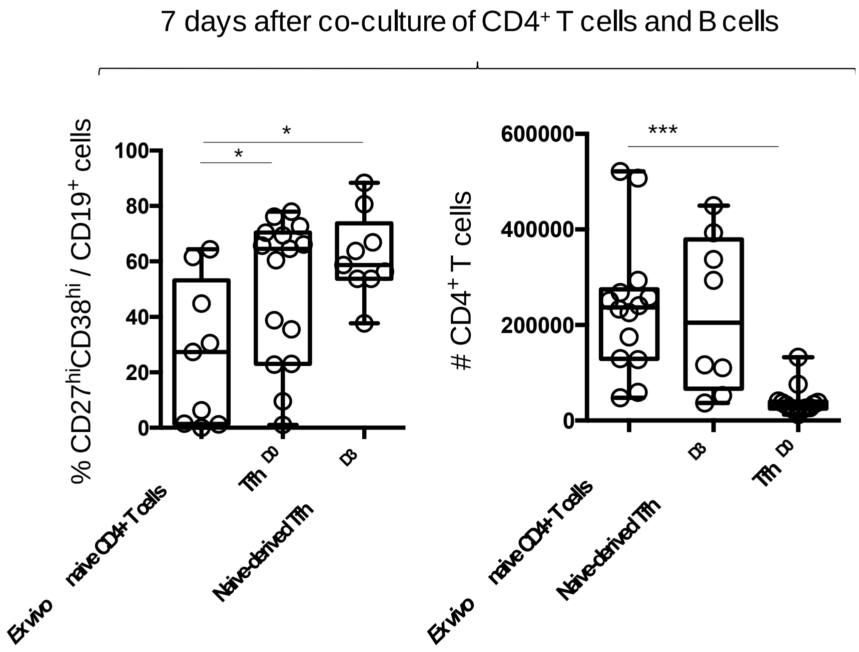

Figure S5. B cell maturation after 7 days of co-culture depends on the quality of CD4^{+} T cells rather than their quantity, related to figure 4F. *Ex vivo* cells (naive and Tfh^{D0}) or naive-derived Tfh^{D3} were co-cultured with autologous B cells for 7 days. Box plots represent the absolute number of CD4^{+} T cells (left) and percentage of CD27hiCD38hi plasma cells among CD19+ cells obtained (right) after 7 days of the T-B cell co-culture (n = 9). A Wilcoxon matched paired test was performed, *p<0.05, **p<0.005, ***p <0.001.

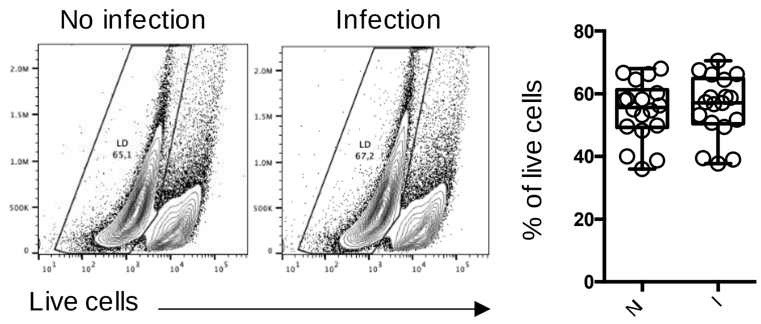

Figure S6. HIV infection does not lead to an increase of cell death at 3 days of stimulation, related to figure 5. Representative flow plots showing the gating strategy for live cell selection after splenocyte stimulation in the absence or presence of HIV infection (right) and the percentage of live cells (left). (20 dot plots as n = 5 individual donors for which the experiment was done 4 times).
